# Don’t Fire Together: Desynchronised Communication Scheduling for Bandwidth-Limited Multi-Robot Exploration

**DOI:** 10.3390/s26154778

**Published:** 2026-07-27

**Authors:** Rong Zhao, Haohua Que, Jiajun Sun, Jiayue Xie, Qian Zhang, Fei Qiao

**Affiliations:** 1 School of Computer Science and Technology, North University of China, Taiyuan 030051, China; 20240008@nuc.edu.cn; 2 Institute for Electronics and Information Technology in Tianjin, Tsinghua University, Tianjin 300467, China; 3 Sense Lab, Department of Electronic Engineering, Tsinghua University, Beijing 100084, China; haohuaque@outlook.com (H.Q.); jiajuns757@gmail.com (J.S.); xiejiayue2004@outlook.com (J.X.); zhangq22@mails.tsinghua.edu.cn (Q.Z.)

**Keywords:** multi-robot exploration, bandwidth-limited communication, event-triggered communication, desynchronised scheduling, communication synchronisation, LiDAR, ROS 2

## Abstract

Cooperative multi-robot exploration relies on a shared belief over teammates’ poses and maps, but bandwidth-limited radios refresh that belief only intermittently. We identify a failure mode beyond simply sending fewer messages: when robots broadcast on a common schedule, the shared belief goes stale *in phase*, the learned explorer acts on the same outdated information across the team, and robots herd toward the same frontiers. We call this the *synchrony tax* and show it is controllable at test time, without retraining. A scheduling layer over a frozen graph-attention policy fixes each robot’s broadcast rate and changes only the *phase* of transmissions, cutting travel by 9.8% and sensing overlap by 13.2% (independent replications reach −12.9%). The primary contributing factor is a higher *peak* (not mean) team staleness, which a staleness-to-overlap bound links to redundant coverage; the benefit accordingly disappears on planners that barely use the shared belief. OW-Desync, a budget-constrained activation policy, carries even staggering to range-limited, heterogeneous channels, improving the freshness it controls while matching staggering on the task. Four-robot experiments over real WiFi reproduce the timing-to-staleness stage of the mechanism at a matched rate: across five repeated paired trials, staggering cuts the measured peak team staleness by 37% (all pairs concordant, within 1.5% of the predicted optimum) with the mean unchanged.

## 1. Introduction

Teams of mobile robots carrying onboard LiDAR and running real-time LiDAR-inertial odometry [1,2] can explore large unknown environments far faster than a single robot, in applications from search-and-rescue to inspection and subterranean mapping [3,4]. What limits such a team is rarely individual sensing. It is the freshness of the *shared belief* each robot holds about its teammates’ poses and maps, which has to travel over a wireless link of limited and contended bandwidth [5,6,7]. How a team spends that scarce link, how often and *when* each robot shares its state, is a first-class sensing-system design question and the subject of this paper.

Learned explorers make the question acute. State-of-the-art policies such as ARiADNE [8] and its bandwidth-limited extension [9] exchange a learned message at *every* planning step [4,8,9], which onboard radios with finite bandwidth and packet loss cannot sustain. Throttling communication is the obvious fix, but it exposes a failure mode that has little to do with sending fewer messages. When a budgeted team broadcasts on a *common* schedule, every robot’s view of its teammates goes stale at the same moment; the learned policy then acts on correlated, out-of-date beliefs, so the robots choose the same frontiers, cover ground twice, and travel further than they need to. We call this the *synchrony tax*: limited bandwidth forces synchronised updates, synchronised updates correlate belief staleness, and correlated staleness turns into duplicated frontier choices and wasted travel.

The remedy is to treat *phase* as a resource. At a fixed per-robot broadcast rate, *when* each robot transmits relative to the others is as controllable as how much it sends, and far cheaper to change. We show that communication timing is an overlooked control knob: at the same nominal broadcast rate, changing only the phase of transmissions removes a substantial efficiency penalty that synchronised schedules pay under scarce communication. This is naturally read in the language of information freshness. A robot acts on the last update it received, whose error grows with its age, so the team pays for coordination through how stale its shared belief is at decision time. Age-of-Information theory shows that on a shared or interference-limited medium this freshness is set by *when* sources transmit relative to one another, and that spreading correlated sources in time is close to optimal [10,11,12]. Because the scheduler runs on a frozen policy at test time, it needs no retraining and adds sub-millisecond overhead per step at N=8 (a few milliseconds at N=20), so it applies to an already-deployed explorer.

The novelty of this paper is that it identifies broadcast phase as a coordination resource that communication-limited multi-robot learning has left largely unexamined; explains its effect through a single supported mechanism (the peak, not the mean, of team staleness); shows that exploiting it is free (a task-level gain at a matched per-robot rate in simulation, whose timing-to-staleness stage is reproduced quantitatively on four robots over real WiFi); and charts its boundary: the benefit appears exactly where coordination runs through a shared belief, plain even staggering already attains the bound optimum on a symmetric medium, and OW-Desync carries the same principle to heterogeneous, interference-limited channels. Our contributions are as follows:

1. **The synchrony tax.** At a fixed per-robot broadcast rate, changing only the *phase* of transmissions cuts travel and sensing overlap by 9.8% and 13.2% on the learned explorer (independent replications of the same condition reach −12.9% and −14.4%), largest at the scarcest budget tested. This is a test-time phenomenon that needs no retraining, and desynchronised schedules in fact send up to 16% more bytes, so the saving is not explained by the nominal broadcast rate or realised byte savings (Section 4).
2. **A staleness mechanism.** The cost is not a worse *average* age but a higher *peak* team staleness. Synchronised schedules make all beliefs stale together; even staggering lowers that peak while preserving its mean, which a staleness-to-overlap bound links to redundant sensing (Section 3.4). The benefit disappears on planners that barely deconflict through the shared belief (with a small apparent cost for the most myopic one), which is the disappearance the mechanism predicts. Four-robot WiFi experiments reproduce the timing-to-staleness stage of the mechanism on hardware: at a matched rate, staggering cuts the measured peak team staleness by 34–37% across six paired runs on two fleets (five repeated trials: all pairs concordant, paired *t* $p<10^{-7}$), close to the no-jitter optimum, with the mean unchanged (Section 4.9).
3. **From even staggering to OW-Desync.** Even staggering is the natural schedule on a symmetric shared medium; on a range-limited, heterogeneous interference graph we derive **OW-Desync**, a budget-constrained activation policy that generalises it [11,13]. OW-Desync is a principled extension of the mechanism to lossy, heterogeneous channels; it provably improves the freshness objective it controls and, on the exploration task, *matches* even staggering rather than beating it, so the task-level gains come from desynchronisation itself rather than from OW-Desync specifically (Section 3).

Throughout, the scheduler is a test-time layer over a frozen explorer, not a new exploration policy.

## 2. Related Work

### 2.1. Communication-Constrained Multi-Robot Exploration and Real-Robot Systems

Autonomous exploration was classically driven by frontier selection [14], coordinated frontier assignment for teams [4], and sampling-based trees [15]; deep reinforcement learning later addressed cluttered single-robot exploration [3] on standardised benchmarks [16], and learned map prediction accelerates exploration further by inferring unseen structure from the partial map [17,18]; for teams, attention-based graph policies (ARiADNE [8]) and asynchronous multi-agent RL [19] now lead. A privileged-learning variant explicitly targets *bandwidth-limited* multi-robot exploration [9], and we use this as the frozen backbone for which we schedule communication; such systems share learned messages frequently and largely assume an abundant channel, which is the gap we address. Classical, non-learned pipelines have long treated communication as a constraint on coordination: coordinated frontier assignment [4], planning under periodic-connectivity requirements [20], information-theoretic control of distributed sensing [21], and hierarchical decentralised exploration robust to limited communication [22] constrain *where* robots may go so that the channel suffices, and the survey of [23] maps this landscape; we instead fix the budget and control *when* it is spent. Orthogonal per-robot constraints, such as energy-efficient legged locomotion and foothold planning [24], compound these coordination costs on physical platforms. Closely related communication-limited exploration work reduces what is sent (e.g., sharing only positions) or plans intermittent rendezvous [25]; we instead hold the per-robot broadcast rate fixed and control *timing*. The same communication pressure appears in real robotic stacks built on LiDAR odometry/mapping (LOAM [1], LeGO-LOAM [26], LIO-SAM [27], 2D SLAM [28], small-FoV LiDAR odometry [29], FAST-LIO2 [2]) and multi-robot SLAM (DOOR-SLAM [5], Kimera-Multi [6], and centralised collaborative dense TSDF mapping [7]), which motivates bandwidth-aware coordination when deployed on ROS 2 [30] with Nav2 [31].

### 2.2. Information Freshness and Communication Scheduling

Age-of-Information minimises staleness under an update budget [32,33,34]; on a symmetric shared medium, a greedy max-age-first/round-robin rule is age-optimal, while general networks call for Whittle-index restless-bandit schedulers [10,35], and value-of-information weights an update by its task impact [36,37]. Transmitting only on sufficient change is the event-/self-triggered-control idea [38,39,40,41,42], of which our drift-triggered broadcast gate is one self-desynchronising instance. Two structural results anchor our method. First, for *correlated* sources even simple staggering is *near-optimal*: round-robin is provably within a small constant of optimal [43] and correlation-aware scheduling cannot beat correlation-agnostic age scheduling by more than a constant factor [12,44]; this is why, in our symmetric regime, our Proposition 3 (evenly spaced offsets $\phi_{k}=\left\lfloor \left(k-1\right)T/N\right\rfloor$ minimise peak staleness) is hard to beat. Second, on an *interference graph* where only an independent set may transmit per slot, a stationary randomised activation policy is exactly peak-AoI-optimal [11]; OW-Desync instantiates this for the multi-robot-exploration overlap cost with heterogeneous weights and a per-slot budget, recovering even staggering as the symmetric special case. Decentralised self-organising desynchronisation (DESYNC [13], recently demonstrated on a drone swarm [45]) realises evenly spaced broadcasts from local overhearing with no coordinator. Our contribution is to carry this freshness-scheduling theory from a networking objective to a robotics *task* cost (sensing overlap and travel) under a frozen learned explorer.

### 2.3. Learned and Test-Time Communication on Frozen Policies

Cooperative multi-agent RL learns *what*, *whether*, and *with whom* to communicate. Representative frameworks include differentiable inter-agent learning [46], CommNet [47], BiCNet [48], ATOC [49], I2C [50], MAGIC [51], and TarMAC [52] atop centralised-training value factorisation [53,54] and attention [55]. The most relevant are budget-aware methods: SchedNet [56], IC3Net [57], When2com [58], decomposability/information-bottleneck compression [59,60], event-triggered MARL [61], value-of-information gating [62], model-based communication that skips predictable messages [63], and a recent survey [64]. These learn the communication policy end-to-end; we instead provide a lightweight, test-time scheduling *layer* for a *frozen* explorer and isolate *synchronisation* as the dominant cost. We separate two orthogonal axes of communication economy: *how much* to transmit (rate/volume, the target of the above) versus *when* at a fixed broadcast rate (phase/synchronisation, ours), and our baselines instantiate prior paradigms: periodic = time-triggered, distance-triggered = event-triggered [40], staleness-greedy = Age-of-Information [32], round-robin/random = structured/naive budgeted schedules [56].

## 3. Materialsand Methods

### 3.1. System Model and Problem

**Figure 1:**
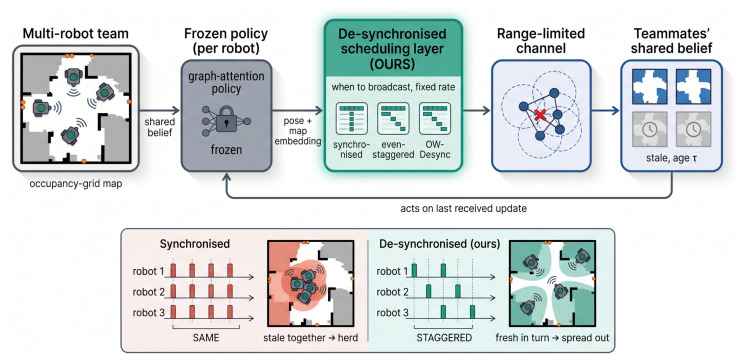
System overview. A frozen, pretrained multi-robot explorer (**left**) plans against a shared belief of teammates’ poses and map embeddings. We insert a test-time desynchronised scheduling layer that decides, at a fixed per-robot rate, *when* each robot broadcasts (synchronised, even-staggered, or OW-Desync) over a range-limited, lossy channel; this sets how stale each teammate’s belief is, and hence travel and sensing overlap. Bottom: Broadcasting on a common schedule (**left**) aligns the robots’ updates, so their beliefs go stale together and they herd onto the same frontiers, whereas desynchronising the phase at the *same* rate (**right**) staggers the updates, so the team stays fresh in turn and spreads out. This wasted-effort gap is the *synchrony tax*.

Figure 1 summarises the setup. *N* robots explore an unknown 2-D environment; robot *i* holds a belief occupancy map and a 64-dimensional learned state embedding produced by the frozen policy [9]. Coordination relies on each robot’s pose and embedding, shared over a bandwidth-limited channel. Both the pose and the embedding travel on this gated channel, and there is no free side-channel that silently keeps poses fresh. For this reason the *schedule*, rather than the message *volume*, governs exploration efficiency. On a silent link the receiver must act on a stale pose, so the *timing* of refreshes changes *where* robots go, and not just *how many* bytes they send. We consider two budget models: (i) an average broadcast rate (a robot either broadcasts to all or stays silent), and (ii) a directed-link budget of *B* transmissions per step among the L=N(N−1) ordered robot pairs (point-to-point). In the stationary OW-Desync analysis this appears as an average activation budget $\sum_{i}f_{i}\leq B$; round-robin and greedy link schedulers use the corresponding hard per-step cap where stated. On a silent link the receiver retains the last received (stale) pose/embedding. Because coverage saturates, the objective is efficiency: mincommunications.t.coverage≥τ; equivalently mintravel/overlap at a fixed budget.

#### Communication Accounting

Three notions are easy to conflate, so we separate them. The *nominal rate* is how often a robot is scheduled to broadcast (once per period *T*, i.e., N/T transmissions per step); this is what the phase-only experiments hold fixed. The *realised byte volume* is the payload actually sent, which can vary with map and update content, and which we report separately rather than constrain. The *broadcast model* (i) has one sender reach the whole team, whereas the *directed-link model* (ii) caps point-to-point messages and underlies the range-limited interference setting of OW-Desync (Section 3.4). Unless stated otherwise the main experiments compare schedules at a matched *nominal broadcast rate* and vary only *phase*; we do not force every byte to match, and in fact desynchronisation sends up to 16% more bytes, which only sharpens the conclusion that the controllable cost is timing.

### 3.2. Communication Schedulers

We compare four broadcast schedulers, all operating on the *frozen* exploration policy at test time. Each robot *i* either broadcasts its current pose and embedding to the team or stays silent; on a silent step every receiver keeps the last copy it received and acts on that stale state. The schedulers differ only in *when* robots broadcast, and they are matched to the same nominal per-robot broadcast rate. The two periodic conditions transmit exactly once per period; the random and event-triggered conditions are calibrated to the same rate in expectation and over the aggregate evaluation. Thus, differences in exploration efficiency are attributable to broadcast *timing* rather than to systematic differences in how often robots transmit. Realised byte volume is reported and discussed in Section 4.

#### 3.2.1. Synchronised Periodic (Control)

Every robot broadcasts together every *T* steps, in lockstep. This is the textbook time-triggered schedule [40] and serves as the control: all robots refresh at the same instant, so the team’s beliefs go stale and are refreshed in phase.

#### 3.2.2. Staggered Periodic (Desynchronised)

Each robot still broadcasts once per period *T*, but robot *k* (k=1,…,N) is given a balanced, as-even-as-possible phase offset $\phi_{k}=\left\lfloor \left(k-1\right)T/N\right\rfloor \in \left\{0,\ldots ,T-1\right\}$; when T<N, this balances how many robots share each slot rather than giving distinct offsets. The per-robot rate (number of transmissions per robot) is identical to the synchronised control; only the broadcast *phase* differs. Realised byte volume is *not* held identical: desynchronisation transmits slightly *more* bytes (Section 4), so any efficiency gain cannot be bought with bandwidth.

#### 3.2.3. Random (Desynchronised)

Each robot broadcasts independently, with a fixed probability chosen to match the periodic rate. Independent draws make the team’s broadcasts unlikely to coincide, giving a desynchronised schedule without any imposed phase structure.

#### 3.2.4. Event-Triggered

A robot broadcasts when its state, measured by the drift of its pose and embedding since its last broadcast, exceeds a threshold, otherwise it stays silent [38,40]. Because each robot’s drift crosses the threshold at its own time, this is a principled, self-desynchronising trigger; the threshold is calibrated to the matched budget.

Algorithm 1 summarises one step for the periodic family; random and event-triggered schedulers replace the firing predicate (phase match) with a Bernoulli draw or a drift test, respectively. The exploration backbone is frozen throughout: only the schedule changes, never the policy.
**Algorithm 1** Matched-budget broadcast scheduling (one step at time *t*)**Require:** 
robots {1,…,N}; period *T*; phase offsets $\left\{\phi_{k}\right\}$ ($\phi_{k}\;=\;0\;\forall k$ synchronised, $\phi_{k}\;=\;\left\lfloor \left(k-1\right)T/N\right\rfloor$ staggered)1:$F\leftarrow \{\;k:\left(t-\phi_{k}\right)\;\mathrm{mod}\;T=0\;\}$       ▹ robots whose broadcast slot is due2:**for all** k∈F **do** broadcast $\left(p_{k},\;{\mathrm{embedding}}_{k}\right)$ to all receivers; ${\hat{p}}_{j}^{\left(k\right)}\;\leftarrow \;p_{k}\;\forall j$3:**end for**4:**for all** receivers *j* and silent senders k∉F **do** keep stale ${\hat{p}}_{j}^{\left(k\right)}$▹ act on last received state5:**end for**

#### 3.2.5. Derived Closed-Loop Schedulers, and Why Even Staggering Is the Near-Optimal Default

The schedules above are open-loop. One can instead act on the staleness → overlap bound of Section 3.4: since that bound is the contention-weighted team staleness $\sum_{ij}w_{ij}\tau_{ij}$, the greedy step refreshes the robots that are simultaneously *stale* and *contending*, broadcasting at the periodic budget the robots of largest index $\tau_{i}\cdot c_{i}$, where $\tau_{i}$ is staleness and $c_{i}=\sum_{j\neq i}\exp\left(-{∥{\hat{p}}_{i}-{\hat{p}}_{j}∥}^{2}/2\sigma^{2}\right)$ is contention from believed teammate positions, the Age-of-Information max-weight/Whittle-index policy [10,44] cast as a desynchronisation rule. We likewise evaluated a joint-submodular max-coverage scheduler and a value/loss-aware Whittle index. In the homogeneous limit all three reduce to refreshing the stalest robot in turn, i.e., evenly spaced phase staggering, which Proposition 3 proves bound-optimal. On our maps *none* of these sophisticated schedulers significantly improves on plain even staggering (Section 4), exactly as correlated-source Age-of-Information theory predicts [12,43]: on a symmetric single broadcast medium, uniform staggering is within a small constant of optimal, so added scheduler complexity cannot pay off there. We therefore adopt even staggering as the practical default in the symmetric regime, and treat this null as positive evidence for its near-optimality.

#### 3.2.6. OW-Desync: The Optimal Schedule for a Range-Limited Interference Channel

**Figure 2:**
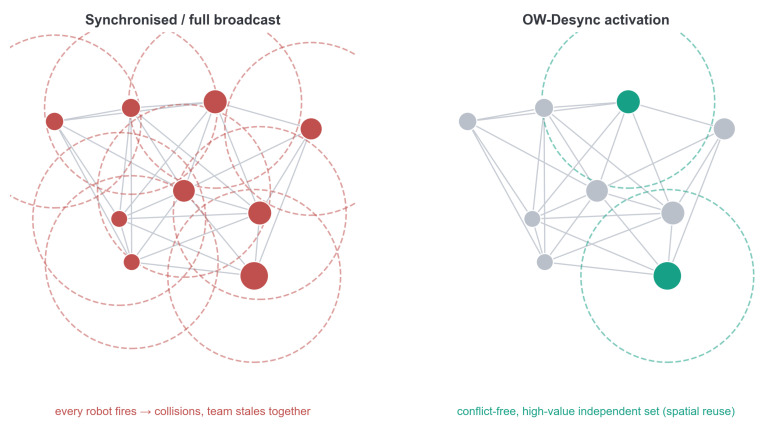
The range-limited interference channel and OW-Desync activation. (**Left**) Synchronised or full-mesh broadcasting fires every robot in the same slot, so co-located transmitters (dashed range rings) collide and the whole team’s beliefs go stale together. (**Right**) OW-Desync transmits, each slot, a conflict-free *independent set* of the interference graph, preferring high-value robots (marker area ∝ weight $w_{i}$), while non-interfering robots reuse the medium spatially. Red nodes denote simultaneous, mutually interfering transmissions; teal nodes form the activated independent set; grey nodes are inactive. Dashed circles show communication/interference range, and solid grey edges join interfering robot pairs. When the graph is complete and robots are symmetric, this reduces exactly to even staggering.

**Figure 3:**
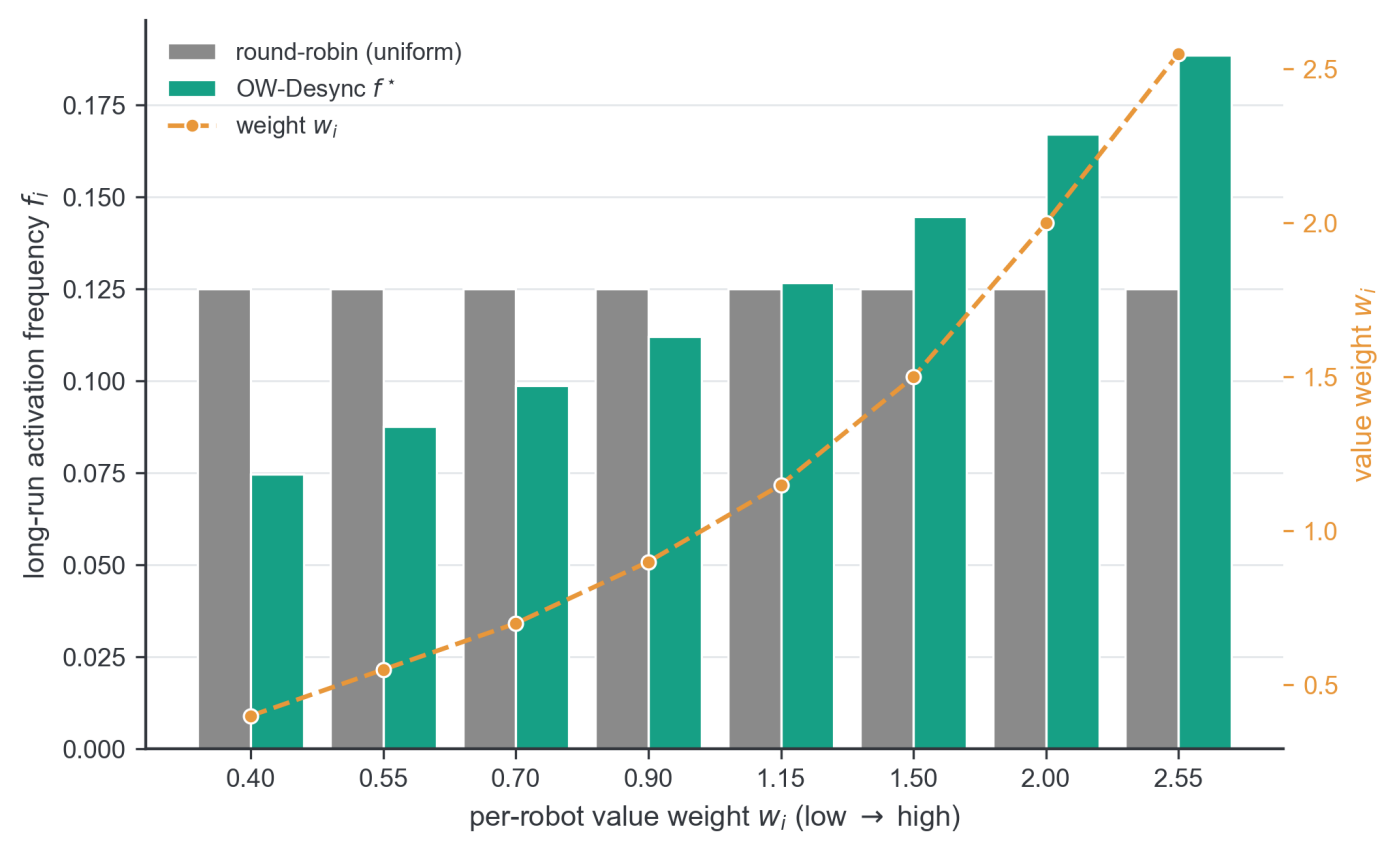
How OW-Desync works in the frequency domain. On a shared medium the convex program assigns each robot a long-run activation frequency $f_{i}^{★}\;\propto \;\sqrt{w_{i}/\gamma_{i}}$ (water-filling), giving more airtime to high-value robots (teal) than the uniform round-robin allocation (grey), which lowers the weighted peak Age-of-Information $A_{p}$. The reduction grows with weight heterogeneity as shown later in the heterogeneity sweep; when weights are symmetric, $f^{★}$ is uniform and OW-Desync reduces to even staggering.

Real radios have finite range: a broadcast reaches only receivers within $R_{\mathrm{comm}}$, and two nearby transmitters that fire in the same slot collide. The feasible transmissions per slot are then an *independent set* of the interference graph *G* (edge (i,j) iff robots i,j interfere; Figure 2), and robots are *heterogeneous*: a robot with a larger sensor footprint or richer local frontier carries more team value, giving a per-robot weight $w_{i}$ (overlap-coupling × informativeness) and channel reliability $\gamma_{i}$. Writing $f_{i}$ for robot *i*’s long-run activation frequency, the weighted peak Age-of-Information $A_{p}$ and the OW-Desync schedule are

(1) $$A_{p}\left(f\right)=\sum_{i}\frac{w_{i}}{\gamma_{i}f_{i}},\;\;\;f^{★}=\arg\min_{f\in P(G,B)}A_{p}\left(f\right),$$

where P(G,B) is the budget-scaled stable-set polytope of the interference graph *G*, so the program is convex. It is realised by a stationary policy that samples independent sets with marginals $f^{★}$ (the policy of Theorem 1); online, we approximate it by transmitting, each slot, the conflict-free maximum-weight set of weight $w_{i}\;\tau_{i}$. When weights and reliabilities are symmetric and *G* is complete (a single shared medium), $f^{★}$ is uniform and OW-Desync *reduces exactly to even phase staggering*; under heterogeneity it serves high-value robots more often (Figure 3) and is provably peak-age-optimal (Theorem 1, generalising the interference-graph optimality of [11]). It thus subsumes even staggering as its symmetric special case and extends it to the realistic interference-limited, heterogeneous setting. The activation frequencies can also be reached *decentrally*, from local overhearing with no coordinator, by a self-organising desynchronisation update [13,45].

### 3.3. Complexity and Overhead

Per step, each scheduler makes *N* independent broadcast decisions, each O(1): a phase test for the periodic schedules, a Bernoulli draw for random, or a state-drift threshold test for the event-triggered gate; memory is $O(N^{2})$ for the per-receiver beliefs and staleness counters. Measured in our CPU implementation, the scheduling step is sub-millisecond (≈0.6 ms at N=8 and a few milliseconds at N=20), which is negligible relative to the team’s per-step graph-attention policy inference and well below typical control periods. The overhead is negligible, and the scheduler runs comfortably onboard in real time. Because the backbone is frozen, no training-time cost is incurred.

### 3.4. Theoretical Analysis

We now make the synchrony-tax mechanism precise: staleness bounds belief error (Lemma 1), correlated staleness inflates the overlap envelope (Proposition 2, which formalises a sufficient mechanism linking staleness to overlap rather than a tight numerical guarantee, as the envelope Φ is only assumed non-decreasing; Φ is deliberately abstract rather than a fitted functional form, the linear envelope Φ(x)=αx being its simplest admissible instance, with its induced empirical relation evaluated in the Results), and spreading broadcasts in time minimises that envelope (Proposition 3 on a shared medium, Theorem 1 on a general interference graph). The proofs are deferred to Appendix A.

**Notation 1.** 
*Time is discrete, t=0,1,…. Robot i has true position $p_{i}\left(t\right)\in R^{2}$. The directed communication links are L={(i→j):i≠j}, with |L|=L=N(N−1); at most B links* deliver *per step (a hard per-step cap, used by the periodic and round-robin schedules; the OW-Desync model of Theorem 1 instead constrains the* average *activation rate, $\sum_{i}f_{i}\leq B$, at the same nominal budget; there, $f_{i}$ counts robot broadcasts, each delivering the robot’s N−1 outgoing links together). Receiver j stores a belief ${\hat{p}}_{j}^{\left(i\right)}\left(t\right)$ of sender i’s position; on delivery of link i→j at step t, ${\hat{p}}_{j}^{\left(i\right)}\left(t\right)\leftarrow p_{i}\left(t\right)$, otherwise it is unchanged. The *staleness* $\tau_{ij}\left(t\right)$ is the number of steps since link i→j last delivered, and the* belief error *is $e_{ij}\left(t\right)=∥p_{i}\left(t\right)-{\hat{p}}_{j}^{\left(i\right)}\left(t\right)∥$. Let $v_{\max}=\max_{i,t}∥p_{i}\left(t\right)-p_{i}\left(t-1\right)∥$ be the maximum single-step displacement and D the environment diameter (so, $e_{ij}\leq D$).*

**Lemma 1** 
(Belief error grows linearly with staleness)**.** *For all i,j,t, $\;e_{ij}\left(t\right)\leq \tau_{ij}\left(t\right)\;v_{\max}$; a stale belief drifts no faster than the robot moves.*

**Proposition 1** 
(Staleness bounds)**.** *Round-robin scheduling (a fixed cyclic order, B deliveries/step) satisfies $\tau_{ij}\left(t\right)\leq \left\lceil L/B\right\rceil -1$ for all i,j and t≥⌈L/B⌉; in particular, any periodic schedule that serves every link once per cycle keeps staleness uniformly bounded.*

**Assumption 1** 
(Deconfliction)**.** *Two robots sense redundantly only when $∥p_{i}-p_{j}∥\leq 2R$ (R the sensing radius); the policy deconflicts using* believed *teammate positions, so a deconfliction failure for a near (“contending”) pair becomes more likely, and not less, as the receiver’s belief error $e_{ij}$ grows. This holds to the extent that the planner actually coordinates on the shared teammate belief: a planner that couples only weakly on that belief violates it, and the scheduling effect should then be weak or absent. This matches what we observe, with desynchronisation strong on the learned graph-attention backbone (which coordinates through the shared belief), weak on the utility frontier, and gone, indeed with a small apparent cost, on the myopic nearest-frontier planner (Section 4).*

**Proposition 2** 
(Overlap bound)**.** *Under Assumption 1, the expected per-step team overlap satisfies*

(2) $$E\left[\mathrm{overlap}\left(t\right)\right]\;\leq \;\Phi \;\left(\sum_{\left(i,j\right)}w_{ij}\left(t\right)\;e_{ij}\left(t\right)\right)\;\leq \;\Phi \;\left(v_{\max}\sum_{\left(i,j\right)}w_{ij}\left(t\right)\;\tau_{ij}\left(t\right)\right),$$

*where $w_{ij}\left(t\right)=⊮\left[∥p_{i}-p_{j}∥\leq 2R+\delta \right]$ marks contending pairs (δ a margin) and* Φ *is non-decreasing.*

**Remark 1** 
(Why desynchronisation helps)**.** *From the bound $\Phi \;\left(v_{\max}\sum_{ij}w_{ij}\tau_{ij}\right)$: at a fixed communication budget the total number of deliveries is fixed, so a* synchronised *schedule makes all $\tau_{ij}$ peak simultaneously (correlated staleness), maximising the instantaneous $\sum_{ij}w_{ij}\tau_{ij}$ during the team’s silent phase and thus the overlap envelope of Proposition 2, whereas* staggering *spreads deliveries so this peak, and hence the overlap, is lower. The argument runs through Assumption 1, so it predicts a benefit only for planners that deconflict on the shared teammate belief: desynchronisation is a property of such learned, belief-coupled coordination rather than a universal law.*

**Proposition 3** 
(Evenly spaced staggering is optimal in the symmetric case)**.** *On a single shared medium in the symmetric, fully contending case (equal, time-invariant link weights with all pairs contending, so every sender carries the same constant outgoing weight), consider periodic broadcast schedules with common period T in which each robot k broadcasts to all others exactly once per period, at the phase-shifted steps $t\equiv \phi_{k}\;\left(\mathrm{mod}\;T\right)$ with offset $\phi_{k}\in \left\{0,\ldots ,T-1\right\}$. Every such schedule has the same per-robot rate and the same broadcast count. Among all offset assignments $\phi =(\phi_{1},\ldots ,\phi_{N})$, the worst-case team-staleness peak $\max_{t}\sum_{ij}w_{ij}\;\tau_{ij}\left(t\right)$, and hence the overlap envelope of Proposition 2, is minimised by the balanced assignment $\phi_{k}=\left\lfloor \left(k-1\right)T/N\right\rfloor$ (exactly optimal when N divides T, and within a(N−1) of a universal lower bound on the peak for general T≥N, with a the common contention weight of the proof; distinct offsets when T≥N, balanced slot loads when T<N) and is maximised by the synchronised assignment $\phi_{1}=\cdots =\phi_{N}$.*

**Theorem 1** 
(OW-Desync minimises the weighted expected peak age on the interference graph)**.** *On an interference graph G where each slot one feasible (independent) set transmits under an* average *per-slot budget B (the constraint $\sum_{i}f_{i}\leq B$ below), with per-robot weights $w_{i}$ and channel reliabilities $\gamma_{i}$, the stationary policy activating independent sets with the marginal frequencies $f^{★}=\arg\min_{f\in P}\sum_{i}w_{i}/\left(\gamma_{i}f_{i}\right)$ of Equation (1), where $P=\{f:\;f=\sum_{S}x_{S}1_{S},\;x\geq 0,\;\sum_{S}x_{S}\leq 1,\;\sum_{i}f_{i}\leq B,$ S independent in G*} *is the budget-scaled stable-set polytope, minimises the weighted* expected *(time-average) peak age $A_{p}=\sum_{i}w_{i}/\left(\gamma_{i}f_{i}\right)$ over all stationary policies on G (and is within a constant factor of all policies [11]). Here, $f_{i}$ is robot i’s long-run* activation *frequency; each activation succeeds independently with probability $\gamma_{i}$, so link i delivers at rate $\gamma_{i}f_{i}$ and, under the stationary geometric-success age model of [11], has time-average peak age $1/(\gamma_{i}f_{i})$.*

Theorem 1 imports its optimisation core from the interference-graph peak-age framework of [11]; we claim no new optimisation theory. Its contribution is the transfer to exploration: task-derived overlap-coupling weights $w_{i}$, an explicit activation budget, and the reduction of Corollary 1, which shows that the exploration-motivated even staggering is exactly the symmetric case.

**Corollary 1** 
(Even staggering is the symmetric special case)**.** *If $w_{i}\equiv w$, $\gamma_{i}\equiv \gamma$ and G is complete (a single shared medium), then $f_{i}^{★}\equiv \min\left(1,B\right)/N$ and OW-Desync coincides exactly with the evenly spaced staggering of Proposition 3; Proposition 3 is thus the symmetric, complete-graph case of Theorem 1.*

**Proposition 4** 
(Strict improvement under heterogeneity, shared-medium case)**.** *In the shared-medium (simplex) case, where the only active constraint is the total budget $\sum_{i}f_{i}\leq B$, if the ratios $w_{i}/\gamma_{i}$ are not all equal, then $f^{★}$ is non-uniform and $A_{p}\left(f^{★}\right)<A_{p}\left(f^{\mathrm{RR}}\right)$ strictly, where $f^{\mathrm{RR}}$ is the uniform (round-robin) allocation, and the gap grows with the dispersion of $w_{i}/\gamma_{i}$. On a general interference graph the optimum is still characterised by the convex program of Theorem 1, without a closed form.*

**Corollary 2** 
(Overlap envelope)**.** *With $w_{i}$ the overlap-coupling of robot i, OW-Desync’s $f^{★}$ minimises the overlap envelope of Proposition 2.*

### 3.5. Experimental Setup

We evaluate on the frozen pretrained checkpoint of [9] over 20 held-out maps, with parallel CPU evaluation. Simulation experiments use Python 3.8, PyTorch 2.3.1, Ray 2.10.0, and SciPy 1.10.1. The hardware software stack uses ROS 2 Humble, the Nav2 Humble distribution, and the FAST-LIO2 repository implementation. Metrics: final coverage ratio, total travel distance, sensing overlap ratio, and communication volume (bytes). Significance is assessed with paired Wilcoxon signed-rank tests and bootstrap 95% confidence intervals over matched (map, seed) pairs. Throughout the figures and tables we mark these paired tests as * for p<0.05, ** for p<0.01, and “n.s.” (not significant) for p≥0.05; a non-significant OW-Desync-versus-even result is the intended “does no harm” outcome. We treat the phase-only control and the matched-budget comparison as the primary tests; the team-size, planner-backbone, correlation, packet-loss, and sensor-range sweeps are exploratory, reported with nominal *p*-values. The two primary tests were specified in advance of the exploratory sweeps, and the headline conclusions rest on them (both remain significant under Holm correction); the individually marginal exploratory points (the 20% packet-loss dip, p=0.057, and the 40 m sensor-range point, p=0.051) carry no conclusion. The decisive phase-only contrast (staggered versus synchronised periodic, N=8, interval 12) recurs across several independently generated batches below (different map/seed draws and spawn layouts): the three-period sweep reported below (n=160 per period), the team-size sweep (n=120), the backbone run (n=100), the ordering check (n=160), and the correlation run (n=160). Its travel effect ranges from −9.8% to −12.9% across these replications; we quote the conservative three-period-sweep values as the headline. The baselines instantiate standard prior paradigms rather than proposed methods. *Original* (every-step broadcast) is the no-throttling reference condition; it is infeasible under the bandwidth budgets studied and is not evaluated. *Periodic* is the textbook synchronised time-triggered control [40], included as the control needed for the desynchronisation comparison and not as a method we advocate. *Staggered* (phase-offset periodic) is our de-sync control; *EventTrig* (distance/event-triggered [38,40]) and *Random* complete the naive fixed-rate schedules; *round-robin* appears as the uniform allocation in the OW-Desync freshness comparison. These fixed-volume schedules connect to Age-of-Information broadcasting, where a max-age-first/greedy-AoI rule is the natural age-based baseline [10,32]. The implementation’s broadcast *interval* and the theory’s *period T* denote the same quantity and are used interchangeably. Throughout, we hold the per-robot broadcast rate fixed (realised byte volume is reported separately) and vary only *when* robots broadcast, isolating schedule structure from bandwidth.

## 4. Results

### 4.1. Synchronisation Is the Controllable Cost

The benefit of desynchronisation first appears even in an approximately byte-matched comparison. At a matched, scarce broadcast budget (N=8, ≈47–50 kB, identical coverage), merely changing the temporal pattern of broadcasts cuts travel by 10.6% and overlap by 10.8% relative to the synchronised periodic control (Table 1). This is the first sign of the synchrony tax: at essentially the same realised bytes, schedules that avoid lockstep updates waste less motion. The next control is the decisive one, isolating phase itself by comparing synchronised and staggered periodic schedules at the same per-robot broadcast rate.

**Figure 4:**
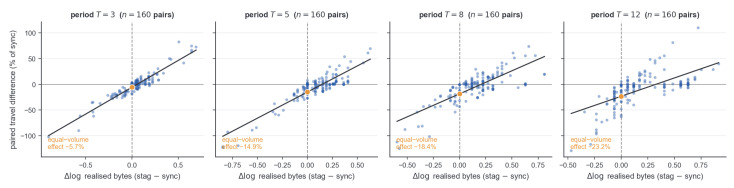
The schedule effect is not a volume effect: each panel plots, for one broadcast period, the paired travel difference (staggered minus synchronised, as % of synchronised travel) against the paired difference in realised log byte volume over n=160 matched (map, seed) episodes. Realised bytes co-vary with travel (positive slopes), which is exactly the confound; the regression intercept at equal realised volume (Δlog bytes =0, orange) remains negative and highly significant at every period (−5.7% to −23.2%, all $p<10^{-14}$), so the desynchronisation gain survives with byte volume held fixed. Grey dashed vertical lines and grey solid horizontal lines mark equal realised volume and zero travel difference, respectively; dark solid lines are least-squares fits, and orange points mark the fitted equal-volume intercepts.

To separate phase from broadcast count, content-adaptivity, and randomness, we run a decisive control. Comparing staggered against synchronised periodic broadcasting at the *same* interval (identical per-robot broadcast rate, the same number of transmissions per robot, differing only in phase) isolates synchronisation from broadcast count, content-adaptivity, and randomness. Staggered wins at every period, with the largest advantage at the scarcest budget tested (Table 2). The two arms are *not* byte-identical: because desynchronisation decorrelates beliefs, each staggered broadcast carries a larger map delta, so staggered in fact transmits *more* bytes (62.0 vs. 53.6 kB at period 12, a 15.7% surplus that itself grows as bandwidth tightens: +6.6%, +13.0%, +15.7% at periods 5, 8, 12). The gain is therefore not a bandwidth *saving*; desynchronisation spends more bytes yet travels less. The surplus could still mean more *delivered* information per broadcast; two controls address this. The byte-matched periodic-versus-random comparison of Table 1 (≈47–48 kB in both arms) reproduces the gain at matched realised volume, and the pooled ANCOVA of the team-size sweep below, which controls for log-communication volume, leaves the effect highly significant. More directly, adding the realised byte volume as a covariate to the paired contrasts (regressing each pair’s travel difference on its log-volume difference) leaves the schedule effect negative and highly significant at every period: the equal-volume effect is −5.7%, −14.9%, −18.4%, and −23.2% of synchronised travel at periods 3, 5, 8, and 12 (n=160 pairs each, all $p<10^{-14}$), so the byte surplus does not account for the gain (Figure 4).

**Figure 5:**
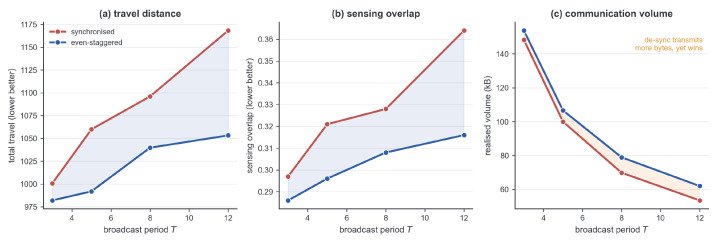
Synchronisation is the controllable cost at a matched per-robot broadcast rate (N=8, periods 3–12). Desynchronised (blue) lies below synchronised (red) in both (**a**) total travel and (**b**) sensing overlap, with the largest gap at the scarcest budget; (**c**) desynchronisation transmits *more* bytes (up to 16% at period 12) yet travels less, so the gain is timing, not a bandwidth saving. The shaded regions show the between-schedule gap: the blue areas in (**a**,**b**) show the efficiency advantage of desynchronisation, and the orange area in (**c**) shows its additional realised byte volume.

Figure 5 summarises the phase-only sweep. At period 12, travel falls by 9.8% and overlap by 13.2% ($p=1.4\times 10^{-5}$ and $1.3\times 10^{-5}$). Bootstrap 95% confidence intervals on the paired difference exclude zero at periods 5, 8, and 12 for both the travel and exploration steps. This is consistent with the byte-matched periodic-versus-random comparison of Table 1 (−10.6% travel, $p<10^{-3}$). Desynchronisation also *accelerates* exploration. At a matched budget the team reaches target coverage in significantly fewer steps. Comparing staggered with synchronised, the bootstrap 95% CIs on the paired step difference exclude zero at periods 5, 8, and 12 (for instance, −1.6 steps, CI [−2.4,−0.8] at period 12). The gain therefore lies in exploration time as well as travelled distance.

### 4.2. Generalisation Across Team Size

**Figure 6:**
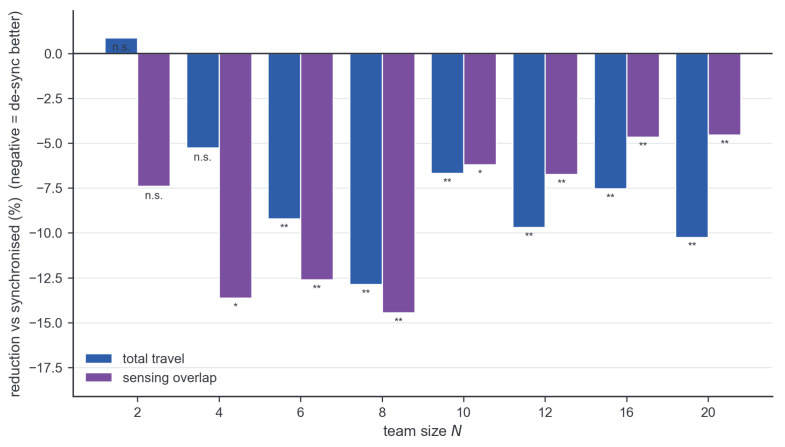
Desynchronisation advantage across team size (N=2–20) from the phase-only control (staggered vs. synchronised periodic at matched budget, interval 12): total-travel (blue) and sensing-overlap (purple) reduction, negative is better. * p<0.05, ** p<0.01; n.s. = not significant.

Repeating the decisive phase-only control (staggered versus synchronised periodic at the same interval, hence matched per-robot broadcast rate) across team sizes from N=2 to N=20 confirms the advantage is not specific to N=8 (Table 3, Figure 6; interval 12, n≥102 paired runs per *N*). The desynchronisation travel reduction is significant (p<0.05) at every N≥6, reaching −12.9% at N=8 ($p=1.6\times 10^{-6}$); it is marginal at N=4 (−5.3%, p=0.07) and absent only for the N=2 dyad, where there is little teammate coordination to de-correlate. Sensing overlap falls at every N≥4 (up to −14.4%). A pooled ANCOVA that additionally controls for log-communication volume and map difficulty agrees (highly significant), and the effect emerges once team coupling is substantial (absent at N=2, marginal at N=4) and is significant for every N≥6 through N=20. Among the desynchronised schedulers, the embedding-drift-triggered gate performs comparably to random staggering, consistent with synchronisation being the first-order effect, with the trigger choice a second-order one.

### 4.3. The Synchrony Tax Appears Only When the Planner Uses the Shared Belief

**Figure 7:**
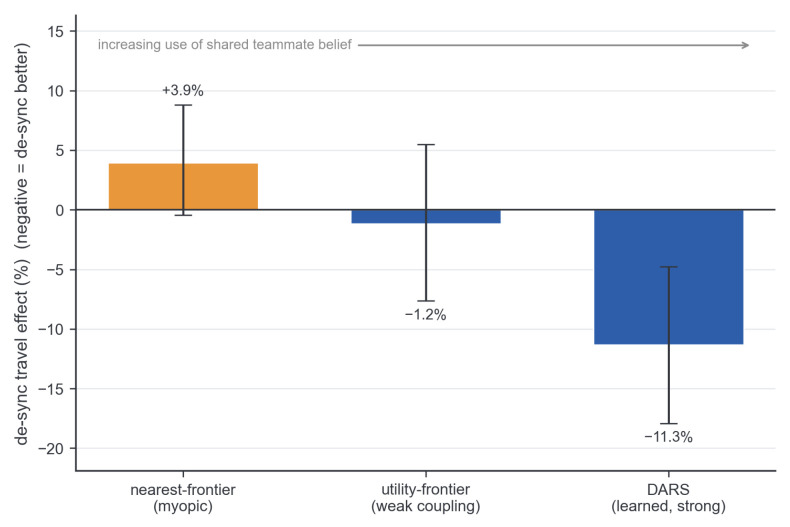
Desynchronisation effect by planner backbone (staggered vs. synchronised periodic, interval 12, N=8; negative means desynchronisation is better). The benefit grows with how much the planner coordinates through the shared teammate belief: harmful for the myopic nearest-frontier planner, weak for the utility frontier, and strong for the learned graph-attention policy. Error bars are 95% bootstrap confidence intervals (the DARS interval excludes zero; the others do not). The paired Wilcoxon test additionally detects a small but significant *harmful* effect for nearest-frontier (+3.9% travel, p=0.011); the bootstrap intervals shown here are wider and are reported separately from those exact *p*-values, which are given in the text.

If the synchrony tax is really about correlated stale *beliefs*, it should vanish on planners that barely use those beliefs. This gives a clean negative control. We ran the same phase-only control (staggered vs. synchronised periodic, interval 12, N=8) on two classical frontier explorers that reuse the identical environment, node graph, and communication layer: a *utility* frontier planner and a myopic *nearest*-frontier planner. The effect tracks how strongly each planner coordinates through the shared belief (Figure 7). On the learned graph-attention backbone (the privileged-learning explorer of [9], abbreviated DARS), staggering cuts travel by 11.3% ($p=2\times 10^{-4}$); on the utility frontier it is only directional (−1.2%, not significant); and on the myopic nearest-frontier planner, which deconflicts only weakly on teammate state, the benefit disappears and a small apparent cost appears (+3.9% travel at interval 12, p=0.011 under the paired Wilcoxon; the bootstrap confidence intervals in Figure 7 are wider and exclude zero only for DARS). This negative control is informative rather than a failure to generalise: the staleness-to-overlap argument predicts the *disappearance* of the benefit when belief coupling is weak (it runs through the deconfliction assumption, Section 3.4); the small residual cost is not predicted by the bound, and we read it as re-planning churn that asynchronous belief updates trigger in a myopic planner, a hypothesis the present experiments do not isolate. We therefore scope the finding to learned, belief-coupled coordination (Table 4).

### 4.4. Mechanism: Desynchronisation Decorrelates Staleness

The staleness-to-overlap bound (Proposition 2) attributes overlap to the *peak* team staleness, not its mean. For a period-*T* schedule, each link’s staleness is a sawtooth, so the team-staleness sum has the *same* time mean, and the schedules issue the *same number of broadcasts*, under synchronised and staggered broadcasting; only the simultaneity differs. Evenly spaced staggering leaves the mean unchanged but roughly halves the peak (a 41–50% reduction across N=4–12 at T=12); the measured sensing overlap falls accordingly (Figure 6). Desynchronisation therefore works by *decorrelating* staleness across robots, not by reducing it. Two further checks separate the peak from the mean. Within each period the mean team staleness is identical across the two schedules by construction, yet overlap falls (Table 2); and across the eight (schedule, period) conditions, jointly regressing mean overlap on the peak and the mean of the team-staleness sum attributes the variation to the peak (p=0.001), with no additional contribution from the mean (p=0.16), as reported below.

**Figure 8:**
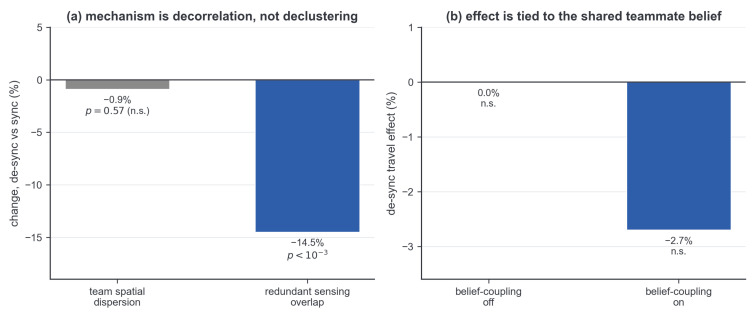
Two controls isolating the mechanism. (**a**) Desynchronisation leaves team spatial dispersion statistically unchanged (−0.9%, p=0.57) while sharply cutting redundant sensing overlap (−14.5%, $p<10^{-3}$): the gain comes from decorrelating stale beliefs, not from physically spreading the robots. (**b**) When the planner is made to ignore the shared teammate belief (deconfliction weight set to zero) the desynchronisation effect vanishes exactly (0.0%); restoring belief coupling restores it only directionally (−2.7%, n.s.), so the causal force rests on the exact null at zero coupling. n.s. means not significant.

Two controls support this causal reading. First, when the planner is made to ignore the shared teammate belief entirely (the utility frontier with its deconfliction weight set to zero), the desynchronisation effect vanishes *exactly* (synchronised and staggered runs are identical, 0.0%); restoring belief coupling restores the benefit only directionally (−2.7% travel at weight 1, stable near −2.8% up to weight 6, n=160 per weight, p=0.56, not significant, consistent with this planner’s weak coupling in Table 4). The causal force of this control rests on the exact null at zero coupling, with the dose direction as corroboration. Second, the benefit is not simple spatial declustering: team spatial dispersion is statistically unchanged between synchronised and staggered runs (p=0.57), while redundant *sensing* overlap falls sharply (−14.5%, $p<10^{-3}$), consistent with decorrelated stale beliefs reducing simultaneous redundant coverage rather than physically spreading the robots (Figure 8).

**Figure 9:**
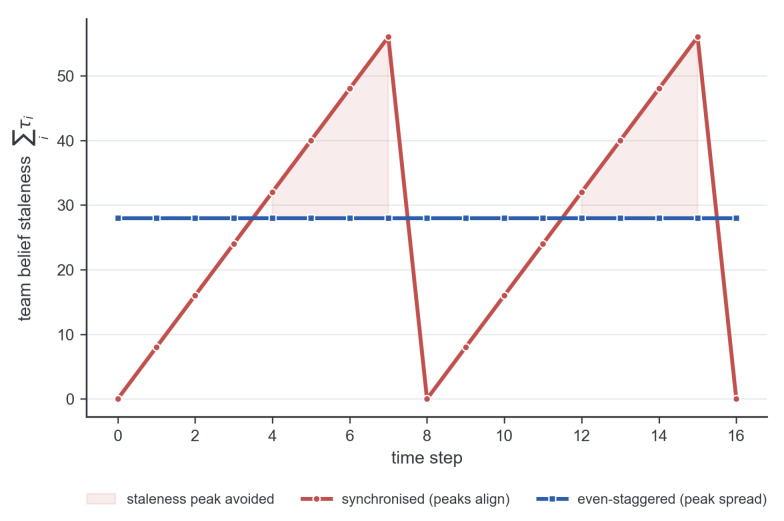
Time-domain view of the mechanism (N=8, period T=8). Each link’s staleness is a sawtooth; under synchronised broadcasting the sawtooths peak together so the team-staleness sum $\sum_{i}\tau_{i}$ swings to a large peak (red), whereas even staggering spreads the offsets and flattens the sum toward its (identical) time mean (blue). The shaded area is the peak the staleness-to-overlap bound penalises and that Proposition 3 minimises.

Figure 9 gives the time-domain view of this decorrelation.

#### Empirical Support for Proposition 2 in the Age-of-Information Metric

**Figure 10:**
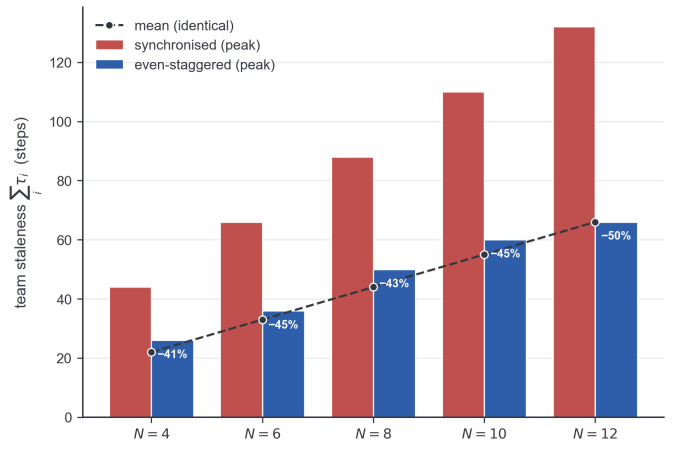
Desynchronisation decorrelates staleness at constant mean. For broadcast period T=12 the team-staleness sum $\sum_{i}\tau_{i}$ has identical time mean (dashed) under synchronised and staggered schedules, but even staggering roughly halves its peak (percentages shown), the quantity the staleness-to-overlap bound penalises.

**Figure 11:**
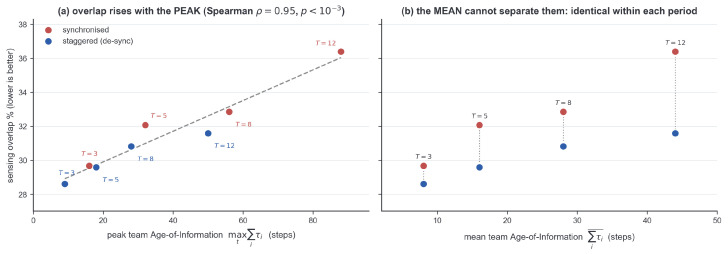
Empirical support for Proposition 2 in the Age-of-Information metric. Each point is one of eight N=8 broadcast conditions (synchronised and staggered periodic, intervals 3,5,8,12). (**a**) Measured sensing overlap rises with the team’s peak Age-of-Information, the peak of the team-staleness sum (Spearman ρ=0.95, $p<10^{-3}$); synchronised schedules occupy the high-AoI end and staggered schedules the low end. (**b**) The same overlap values plotted against the *mean* of the team-staleness sum: within each period the two schedules share one mean by construction (dotted verticals), yet their overlaps differ, so the mean cannot explain the separation of the peak.

The staleness-to-overlap bound is naturally read in the language of Age-of-Information, where the relevant cost is the *peak* of the team-staleness sum rather than its mean. We test the bound empirically in this metric. Figure 10 first shows that staggering reduces this peak while preserving the mean. Across eight broadcast conditions (synchronised and staggered periodic at intervals 3,5,8,12, N=8), the measured sensing overlap rises with the team’s peak Age-of-Information, the peak of the team-staleness sum (Spearman ρ=0.95, $p<10^{-3}$; Figure 11). Synchronised schedules sit at the high-AoI end of this relation and staggered schedules at the low end, consistent with Proposition 2: the quantity the bound penalises is the peak team staleness, and overlap tracks it with measured data in the central metric of the Age-of-Information literature. Even staggering is, by Proposition 3, the peak-AoI-optimal fixed-rate periodic broadcast schedule; for symmetric broadcast this coincides with the AoI-optimal round-robin policy [10].

We also check Proposition 3 directly. Comparing synchronised broadcasting against a suboptimal half-split stagger and the evenly spaced stagger (N=8, interval 12), the desynchronisation travel changes are ordered exactly as the theorem predicts: even staggering (−11.7%, $p<10^{-3}$) < half-split staggering (−9.8%, $p=2\times 10^{-4}$) < synchronised broadcasting (0%, baseline). Even staggering yields the largest benefit, confirming that the evenly spaced offsets are the bound-optimal schedule.

### 4.5. The Benefit Tracks Source Correlation

**Figure 12:**
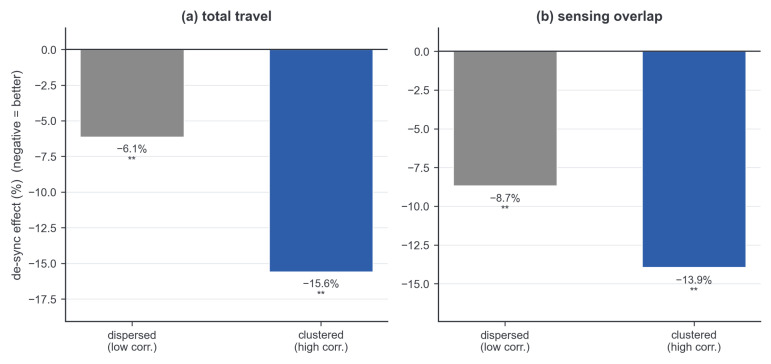
The desynchronisation benefit grows with the team’s initial information correlation, as predicted by [44]. Both (**a**) total travel and (**b**) sensing overlap improve more for clustered, high-correlation spawns (−15.6%, −13.9%) than for dispersed, low-correlation ones (−6.1%, −8.7%) (N=8, interval 12, staggered vs. synchronised periodic; ** p<0.01). More correlated sources gain more from desynchronisation.

If desynchronisation works by decorrelating staleness, its benefit should scale with how correlated the robots’ information is to begin with. The correlated-source Age-of-Information theorem of [44] predicts exactly this: staggering correlated sources in phase helps more when the sources are more correlated. We instantiate that prediction for multi-robot exploration, with redundant sensing overlap as the downstream cost, by varying only the initial correlation of the team. Robots spawned as *clustered* start with highly correlated beliefs and sensing footprints; robots spawned as *dispersed* start with weakly correlated ones. At N=8 and interval 12, the desynchronisation travel benefit is −15.6% ($p<10^{-3}$) for the clustered, high-correlation team but only −6.1% ($p=3\times 10^{-5}$) for the dispersed, low-correlation team (Figure 12). The ordering and its direction match [44]: the more correlated the sources, the more there is to gain from not firing them together (Table 5).

This invites a correlation- or contention-aware scheduler that refreshes the more-correlated robots more often. We tested two and report honestly that neither significantly beat plain even staggering at matched communication. A static rule that separates spatially near robots in phase was, if anything, slightly worse (+3.6% travel, p=0.43). The contention-weighted Whittle-index scheduler of Section 3 was directionally better on heterogeneous (dispersed) teams (−3.2% travel at N=8, p=0.45) but coincided with even staggering for larger teams (+0.7% at N=16, p=0.08) and even for teams with heterogeneous sensing (mixed 10/30 m ranges; −0.6%, p=0.92). Across homogeneous, dispersed, large, and heterogeneous sensing teams, the weighted scheduler never significantly beat even staggering. The reason is structural: for a homogeneously correlated team on a single shared medium, the bound-optimal even spacing of Proposition 3 is already (near-)optimal, leaving little for a weighted rule to exploit. The regime where heterogeneity genuinely pays off is instead the range-limited *interference* channel of Theorem 1: there, OW-Desync provably and measurably improves communication freshness over round-robin (Section 4), while still reducing to even staggering when the team is symmetric. We therefore adopt even staggering as the simple, bound-optimal default for the symmetric medium and OW-Desync as its optimal generalisation under interference and heterogeneity.

### 4.6. Robustness and Breadth

**Figure 13:**
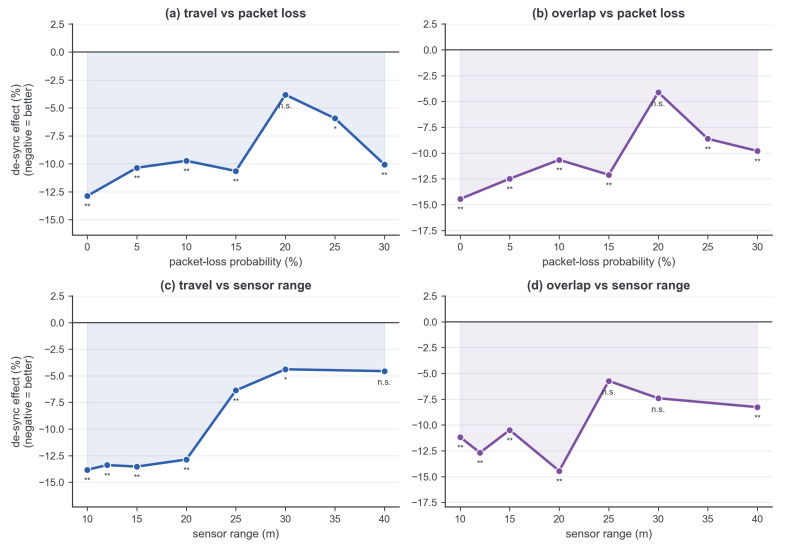
Desynchronisation benefit across two stressors at N=8, interval 12 (staggered vs. synchronised periodic; n≥116 per point). (**a**,**b**) Travel and sensing overlap across seven packet-loss levels (0–30%): the gain persists, the lone shallow point at 20% being consistent with sampling variation flanked by significant neighbours. (**c**,**d**) Travel and overlap across seven sensor ranges (10–40 m): the gain holds across the sweep (the 40 m point marginal, p=0.051) and grows at shorter range, where robots rely more on the shared belief. Shaded areas between each curve and zero visualise the magnitude and direction of the schedule effect. * p<0.05, ** p<0.01; n.s. means not significant.

Two further experiments probe whether the desynchronisation benefit is an artefact of an idealised channel or a single sensing regime. Both use N=8, interval 12, and contrast staggered against synchronised periodic scheduling under paired Wilcoxon tests (n≥116 per cell); both are summarised in Figure 13.

First, we inject independent per-packet erasure as a deliberately simple model of a lossy link; full WiFi contention, retransmission and fading are not modelled here and are deferred to the planned hardware study. We sweep seven loss levels from 0 to 30%. The travel benefit survives; it is −5.9 to −12.9% and significant at every level except 20% (−3.8%, p=0.057), where a shallow dip is flanked by significant reductions at 15% (−10.6%, $p<10^{-3}$) and 25% (−5.9%, p=0.02); the dip is therefore consistent with sampling variation rather than a genuine reversal. Sensing overlap improves in lockstep (−8.6 to −14.5%). The free benefit is thus robust to independent packet loss rather than dependent on a perfect channel.

Second, we sweep seven sensor ranges from 10 to 40 m. The benefit holds across the sweep and grows at shorter range, where the team leans more on the shared belief: travel falls from −13.8% at 10 m ($p<10^{-4}$) to −4.4 to −4.6% at 30–40 m (the 40 m point marginal at p=0.051), and overlap by −7 to −14%. When each robot sees less on its own, decorrelating teammates’ stale views matters more.

### 4.7. From Even Staggering to OW-Desync

#### 4.7.1. Even Staggering Is Near-Optimal in the Symmetric Regime

**Figure 14:**
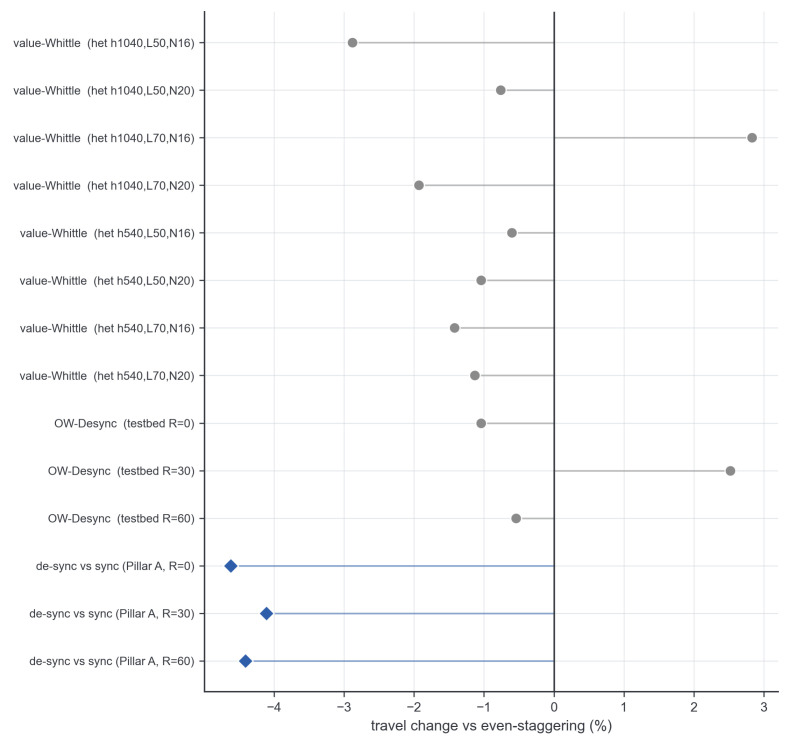
Effect sizes on the exploration objective, measured as travel change relative to even staggering (negative is better). The value/loss-aware Whittle scheduler across heterogeneity and loss regimes, and OW-Desync on the testbed, all cluster around zero (grey; none significantly beats even staggering), whereas the desynchronised-vs-synchronised contrast (blue diamonds) moves travel by −4 to −4.6%. On this objective, scheduling identity is near-optimal; only whether the team is synchronised matters.

We first ask whether a smarter schedule can beat plain even staggering on the exploration task. Across the contention-weighted Whittle, joint-submodular max-coverage, and value/loss-aware Whittle schedulers of Section 3, and a large sweep over broadcast rate (k=N/T∈[0.5,4]), team size (N=4–24), spatial overlap, per-robot value heterogeneity (up to 64×), and packet loss (up to 0.7), *no* scheduler significantly beats even staggering on travel or overlap (e.g., the once-promising joint-submodular overlap effect, −2.9% at n=186, p=0.015, regresses to +0.09% at n=1262, p=0.73). This null is exactly what correlated-source Age-of-Information near-optimality predicts [12,43]: in a symmetric single medium, uniform staggering is hard to beat, so we read the result as positive evidence for its near-optimality rather than a failure (Figure 14).

#### 4.7.2. OW-Desync Is Provably and Empirically Optimal on the Interference Channel

**Figure 15:**
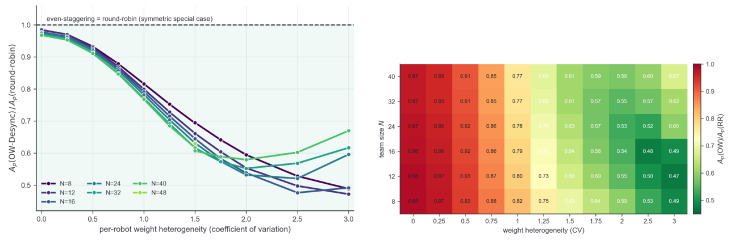
OW-Desync weighted peak-AoI relative to round-robin at matched budget on the interference graph. (**Left**) The ratio is close to 1 (0.97–0.98) at zero weight heterogeneity on this range-limited graph (Corollary 1 predicts exact coincidence on a symmetric shared medium) and falls as per-robot weight heterogeneity grows up to CV≈2, with a partial rebound for the largest teams beyond, for team sizes N=8–48. (**Right**) The same gain as a heatmap over team size and heterogeneity, where every (N,CV) cell with CV>0 improves on average (greener is a larger OW-Desync advantage). The gain is on the communication-freshness objective OW-Desync provably optimises.

**Figure 16:**
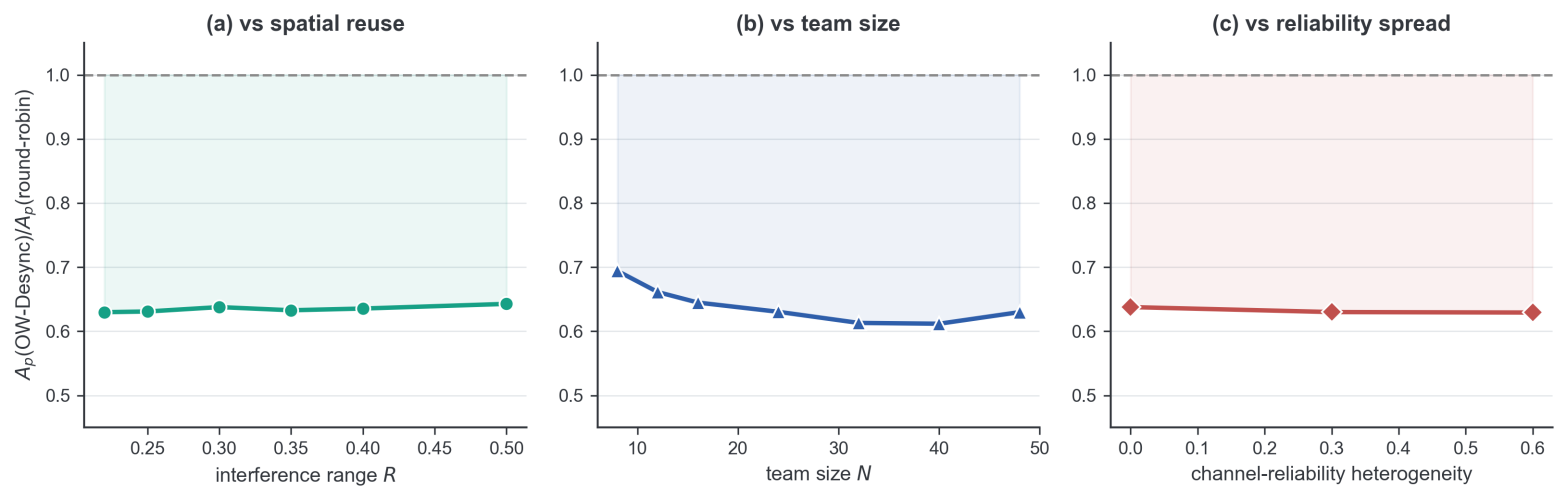
The OW-Desync weighted peak-AoI gain (ratio to round-robin at matched budget; lower is better, dashed line is parity) stays well below 1 across (**a**) interference range/spatial reuse, (**b**) team size N=8–48, and (**c**) heterogeneous channel reliability, so the advantage is not an artefact of any single operating point. The shaded area between each curve and the parity line visualises the magnitude of the OW-Desync advantage.

The picture changes on a range-limited interference graph with heterogeneous robots, where even staggering is no longer optimal (Theorem 1). Measuring the weighted peak Age-of-Information that OW-Desync optimises, the ratio $A_{p}\left(\mathrm{OW-Desync}\right)/A_{p}\left(\mathrm{round-robin}\right)$ equals 1.000 exactly in the fully symmetric complete-graph configuration of Corollary 1; stays at 0.97–0.98 at zero value heterogeneity on the range-limited graph (residual spatial-coupling differences already yield a small gain); and falls with heterogeneity: across *N* mean ratios 0.92, 0.79, 0.64, 0.56 as the weight coefficient of variation grows (0.5 to 2.0; every (N,CV) cell with CV>0 improves on average, with per-instance win fractions 0.80–1.00 over 500 seeds per cell, N=8–48; Figure 15), with a deterministic, realisable activation schedule attaining the convex lower bound. The advantage is robust across team size (N=8–48), spatial reuse, and heterogeneous channel reliability (Figure 16). OW-Desync is therefore Pareto-optimal in the precise sense that it is *optimal* on the communication-freshness axis it controls while *matching* the near-optimal even-staggering baseline on exploration. Practically, OW-Desync earns its coordination machinery once heterogeneity is substantial: in the sweeps of Figure 15, the freshness gain is 8% at a weight CV of 0.5 and 21–44% for a CV of 1–2, while near-homogeneous teams (CV ≈0) gain little and should simply stagger.

#### 4.7.3. On the Frozen Explorer, OW-Desync Preserves Exploration Efficiency

**Figure 17:**
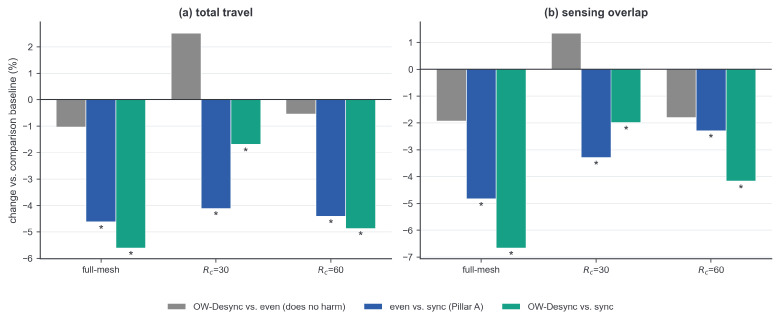
On the frozen explorer under a range-limited channel (N=16, heterogeneous sensing, paired over 240 map–seed pairs per cell). OW-Desync ties even staggering on travel at every communication range (grey, all not significant), while both desynchronised schedules retain a significant advantage over synchronised broadcasting (blue, green; * marks p<0.05), confirming that the synchrony-tax benefit persists on the interference-limited channel.

**Figure 18:**
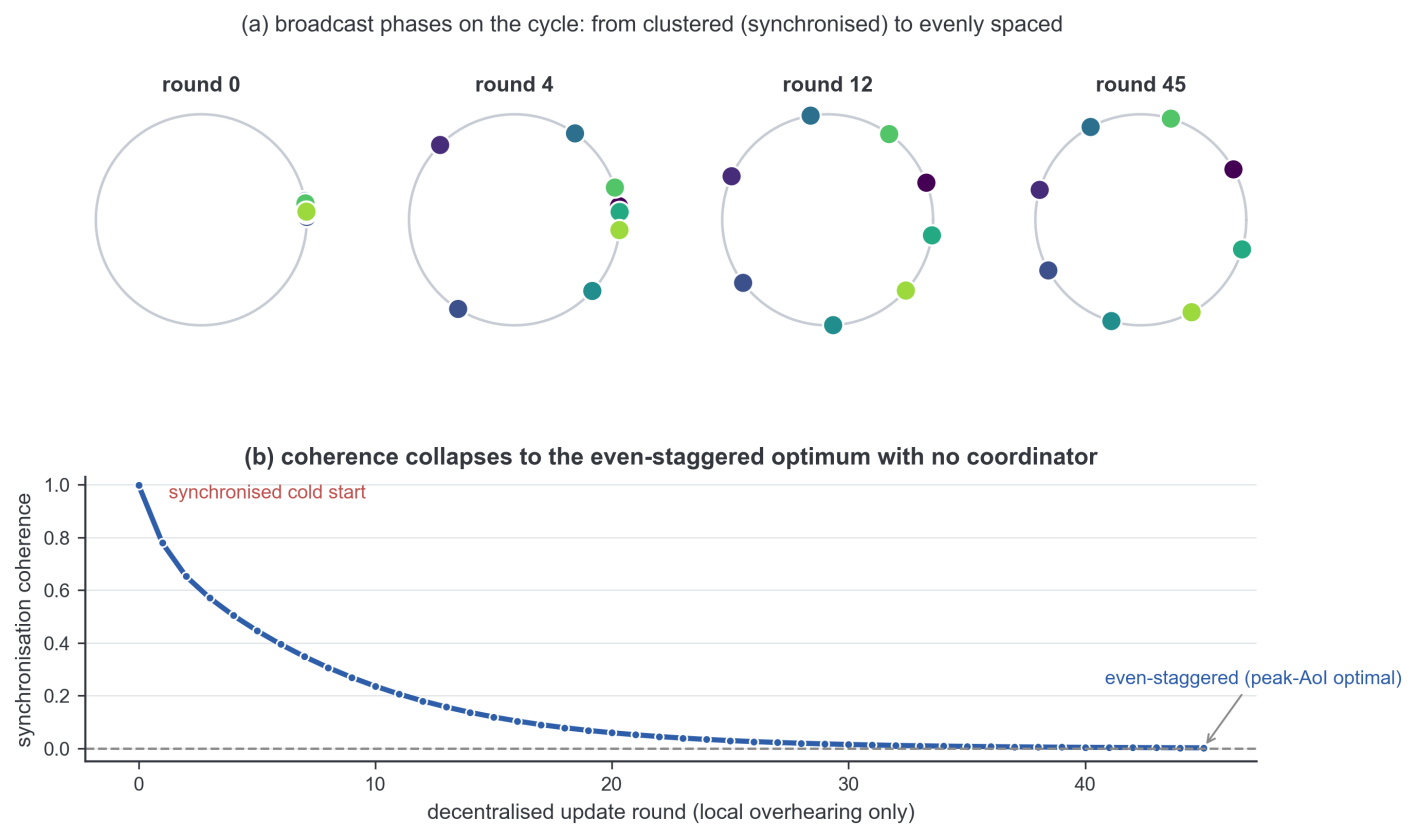
The even-staggered schedule is reachable with no coordinator. Starting from a fully synchronised cold start, a decentralised desynchronisation primitive in which each robot nudges its broadcast phase away from the neighbours it overhears (**a**) drives the phases to even spacing on the broadcast cycle and (**b**) collapses the synchronisation coherence to the even-staggered, peak-AoI-optimal configuration, using only local overhearing (N=8). In (**a**), the eight colours identify the eight robots; in (**b**), the blue curve is synchronisation coherence and the grey dashed line marks the even-staggered optimum.

Running OW-Desync, even staggering, and synchronised periodic scheduling on the frozen explorer under the same range-limited channel (N=16, heterogeneous sensing, paired over 231–240 map-seed pairs per cell), OW-Desync ties even staggering on exploration efficiency at every communication range tested (travel −1.0% to +2.5%, all p>0.05; coverage essentially unchanged, largest change −0.26%), i.e., it preserves exploration efficiency (Figure 17, Table 6). Both desynchronised schedules retain the synchrony-tax advantage over synchronised broadcasting even on the range-limited channel (even staggering vs. sync −4.1% to −4.6% travel, p<0.005; OW-Desync vs. sync up to −5.6%, $p<10^{-3}$). OW-Desync thus inherits the free desynchronisation benefit and adds provably optimal freshness under interference, without trading away exploration efficiency. Its activation frequencies are also reachable decentrally: a self-organising desynchronisation rule recovers a significant fraction of the benefit from a synchronised cold start with no coordinator (overlap −3.0%, p=0.003 at N=16; Figure 18 illustrates the convergence dynamics at N=8).

### 4.8. Qualitative Visualisation

**Figure 19:**
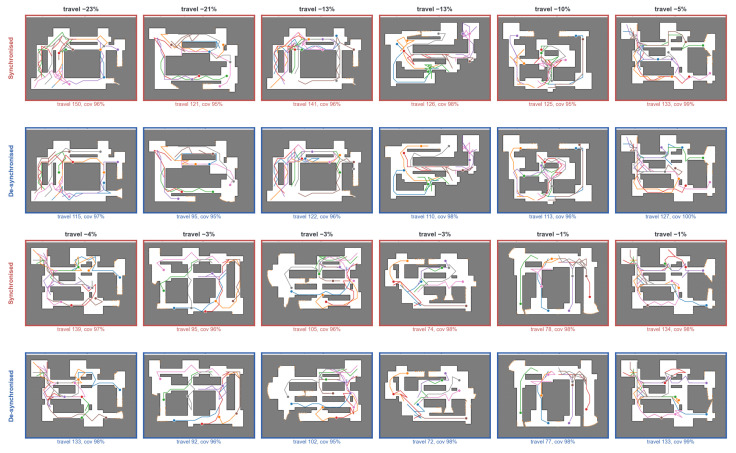
Synchronised (rows 1 and 3, red) vs. desynchronised/even-staggered (rows 2 and 4, blue) exploration at matched bandwidth (N=8, broadcast period T=12); coloured curves are the eight robot trajectories, and the grey/white background is the occupancy map. Desynchronisation cuts herding and redundant travel; each column is one episode (a map and start-layout seed), ordered by the travel reduction printed above each column (−1% to −23%); all reduce travel at essentially unchanged coverage (within 0.3 percentage points, equal or higher in 8 of 12), selected from a 20-map ×3-seed sweep to illustrate the mechanism; aggregate statistics over all maps and seeds are reported above.

Figure 19 contrasts synchronised and desynchronised broadcasting at *matched* bandwidth (N=8, period T=12) across twelve episodes. Under synchronised broadcasting the robots refresh their shared belief simultaneously, briefly act on identical stale information, and herd toward the same frontiers, producing crossing and back-tracking trajectories. Even staggering spreads the belief updates in time, so the team fans out and covers the map with less redundant travel (−1 to −23% here, at essentially unchanged coverage). These episodes are selected illustrations of the mechanism, not a random sample; the aggregate effect over all maps and seeds is quantified by the statistics above.

### 4.9. Hardware Demonstration: The Timing-to-Staleness Stage on Real Robots

**Figure 20:**
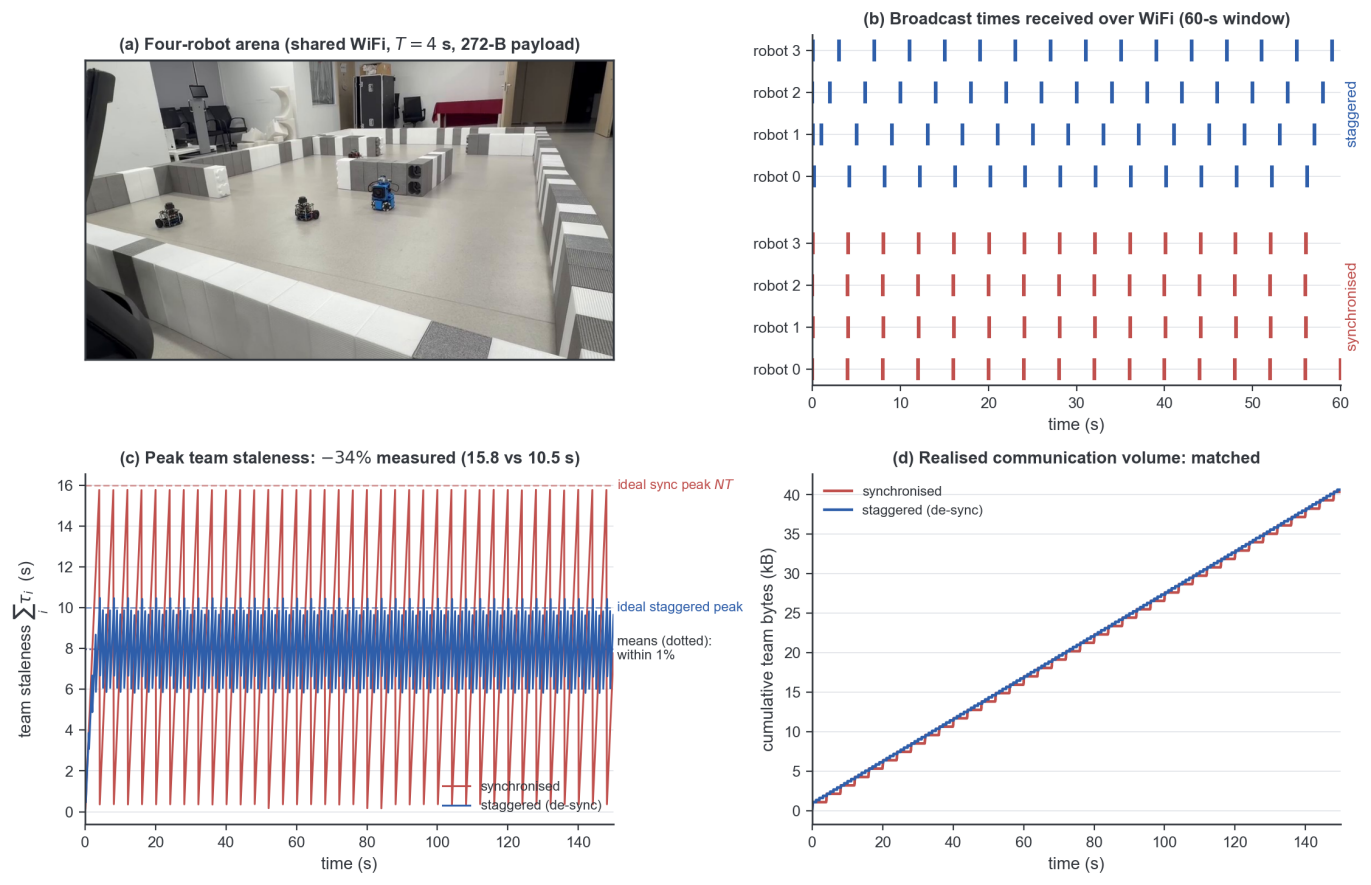
Hardware demonstration of the synchrony-tax mechanism (four robots, real WiFi, one paired trial per condition). (**a**) The walled arena with the four-robot team. (**b**) Broadcast times as received at the central monitor: synchronised (red) robots fire in lockstep; staggered (blue) robots realise even offsets one second apart. (**c**) Team staleness $\sum_{i}\tau_{i}\left(t\right)$: synchronisation drives the peak to the worst case NT while staggering holds it near the no-jitter staggered optimum T(N+1)/2 (dashed), the worst-case-versus-optimum ordering that Proposition 3 formalises; the means (dotted) agree to within 1%, so the difference is purely in the peak. (**d**) Cumulative team communication volume: the two schedules transmit at the same rate with identical payloads, so the curves coincide and the staleness gap is attributable to timing alone.

We deployed the scheduling layer on four differential-drive robots in a walled indoor arena (Figure 20a): a heterogeneous team of two LiDAR-inertial platforms (Mid-360 LiDAR; Livox Technology Company Limited, Hong Kong, China; FAST-LIO2 odometry) and two wheel-odometry Nav2 platforms, all running ROS 2 Humble and sharing one WiFi network. The scheduling layer of Section 3, implemented as a ROS 2 communication node, runs onboard each robot and gates a 272-byte state broadcast (identifier, planar pose, and the 64-dimensional policy embedding) at a fixed period of T=4.0 s (20 ticks of the 0.2 s onboard control loop); a central monitor on the same network records every broadcast it receives, so all timing is measured on one clock after traversing the real channel. We ran one paired trial per condition in the same arena: *synchronised* (all robots on a common phase) versus *evenly staggered* (offsets T/N=1 s apart, i.e., {0,1,2,3} s for robots 0–3), with everything else identical.

**Figure 21:**
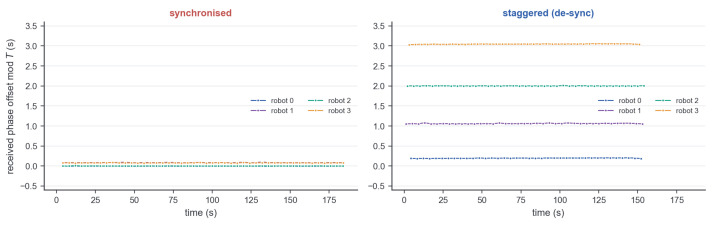
Long-run stability of the hardware schedules: received phase offsets (mod *T*) of every broadcast over the full recordings. The synchronised robots stay aligned (**left**) and the staggered robots hold their assigned offsets (**right**) throughout, with per-robot drift below 5 ms per minute and offset bands within ±15 ms, so asynchronous execution and WiFi jitter did not re-align the schedules over the mission; longer deployments can re-apply the decentralised primitive of Figure 18 online.

Three things transfer from simulation to hardware (Figure 20, Table 7). First, the phase-only control is exact on a physical network: both conditions realise the same per-robot rate (0.0505 vs. 0.0506–0.0519 fires per tick, matched to within 3%) and the identical 272-byte payload, and the cumulative team volume curves coincide (Figure 20d). Second, the schedules survive the real channel: the received broadcast times show the synchronised robots aligned to within about 0.1 s and the staggered robots at offsets {0.20,1.06,2.00,3.05} s, close to the ideal {0,1,2,3} s, WiFi jitter included (Figure 20b). The schedules also hold over the run: each staggered offset stays within a ±15 ms band around its median with drift below 5 ms per minute across the 150 s recording, so no long-run re-alignment was observed; if drift did accumulate on longer missions, the decentralised primitive of Figure 18 re-establishes even spacing online (Figure 21). Third, and centrally, the mechanism appears with the predicted magnitude: the measured peak team staleness is 15.8 s when synchronised versus 10.5 s when staggered, a 34% reduction. Both values sit close to the no-jitter ideals for the per-robot age sum, NT=16 s for synchronised and T(N+1)/2=10 s for even staggering (deviations −1% and +5%), reproducing on hardware the worst-case-versus-optimum ordering that Proposition 3 formalises, while the mean staleness is essentially unchanged (8.0 vs. 7.9 s; unrounded 8.00 and 7.94 s, both within 1% of the schedule-invariant value NT/2=8 s). On hardware, as in theory, synchronisation costs peak freshness and buys nothing in the mean. We emphasise the scope of this demonstration. It is a single paired run, so its numbers are measurements of one realisation rather than statistics, and it establishes only the first stage of the causal chain, from communication timing to peak team staleness, on a physical channel. The downstream stages (staleness to redundant sensing to travel) are quantified in simulation above; the five repeated paired trials reported below add statistical support for exactly this stage.

**Figure 22:**
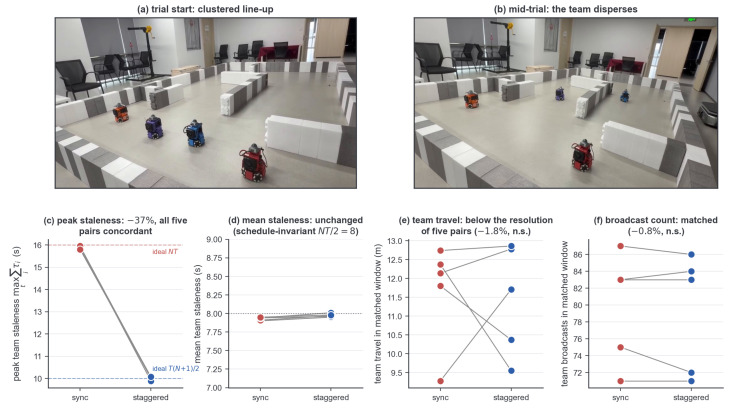
Five repeated paired hardware trials on the second fleet (matched analysis windows). (**a**) Trial start: the four robots begin in a clustered line-up. (**b**) Mid-trial: the team disperses through the arena. (**c**) Peak team staleness: all five pairs fall from the synchronised worst case to the staggered optimum (−37%; dashed lines are the no-jitter ideals NT and T(N+1)/2). (**d**) Mean team staleness: within 0.1 s of the schedule-invariant NT/2=8 s in every run. (**e**) Team travel over the matched window: below the resolution of five pairs (−1.8%, n.s.; the simulation-scale 10% effect needs roughly twenty pairs). (**f**) Broadcast counts: the matched-rate control holds (−0.8%, n.s.).

We then repeated the comparison as *five* paired trials with a second team: four identical mecanum-drive robots with onboard LiDAR-inertial odometry and Nav2 navigation, each broadcasting gated occupancy-map updates (tens of kilobytes per broadcast) at the same period T=4.0 s and logging every signal locally. Each pair ran synchronised (common phase) and evenly staggered (offsets {0,1,2,3} s) in the same arena, and the two runs of a pair are analysed over a common matched window. The mechanism is replicated, now with statistics (Table 8, Figure 22): the measured peak team staleness falls from 15.9 to 10.0 s (−37%), with all five pairs concordant (one-sided exact Wilcoxon p=0.031, the smallest value attainable at n=5; paired *t*-test $p<10^{-7}$) and every run within 1.5% of the no-jitter ideals NT=16 s and T(N+1)/2=10 s; the mean staleness stays within 0.1 s of the schedule-invariant NT/2=8 s in all ten runs; and the broadcast counts are matched (−0.8%, n.s.). The timing-to-staleness stage of the mechanism therefore reproduces across two fleets and two payload regimes (272-byte state vectors and multi-kilobyte map updates) with statistical support. The trials also recorded task-level travel: five pairs resolve the 37% staleness effect decisively but are underpowered for a simulation-scale (≈10%) travel effect against between-run variability (matched-window team travel −1.8%, n.s.); a power calculation indicates roughly twenty pairs would be needed, which we leave to future work.

## 5. Discussion

The main takeaway is that under scarce communication a team pays a *synchrony tax*. When robots can update only intermittently, broadcasting them on a common schedule is the costly choice: their shared beliefs then go stale together, and the explorer acts on correlated, out-of-date information. The lever that removes this cost is timing, not volume. At a fixed nominal broadcast rate, and with desynchronised schedules in fact sending more bytes (up to 16% at the scarcest budget), simply staggering the phase removes a 10–13% travel and overlap penalty paid by synchronised schedules, largest where bandwidth is scarcest. The mechanism is specific. Synchronisation does not worsen the *average* freshness of the shared belief; it worsens its *peak*, and our staleness-to-overlap bound ties that peak to redundant sensing. Even phase staggering provably minimises this peak on a symmetric medium, which is why it, and desynchronised scheduling in general, is hard to beat there.

The synchrony tax is a property of learned, belief-coupled coordination rather than a universal law. A cross-backbone control makes this concrete: the gain is strong on the learned graph-attention explorer, weak on a utility-frontier planner, and gone on a myopic nearest-frontier planner that barely deconflicts through teammate state (with a small apparent cost there that the bound does not predict). Read as a negative control, this disappearance supports the mechanism, a planner that does not act on the shared belief has no synchrony tax to remove, and it also bounds the claim: expect the benefit wherever a policy plans against a shared, communicated belief, and not where robots act independently.

OW-Desync turns this observation into a principled scheduling layer. On a symmetric shared medium, even staggering is already optimal, so there is nothing to add. The interesting regime is the realistic one, where robots have unequal informativeness and the channel is range-limited, so symmetry breaks and the question becomes how to *allocate* freshness across heterogeneous robots under interference. OW-Desync answers this by minimising the weighted expected peak age over the feasible activation polytope: it recovers even staggering as the symmetric special case and improves the freshness objective it is designed to optimise while preserving exploration efficiency on the frozen explorer.

## 6. Limitations and Future Work

The backbone is frozen, so what we contribute is a test-time scheduling layer rather than an end-to-end co-trained system. Because exploration coverage saturates under the budgets we study, the gains we measure are in efficiency (travel and sensing overlap) rather than in final coverage. The synchrony tax is also backbone-dependent: it is a property of learned, belief-coupled coordination, and a planner that does not act on the shared belief sees little or none of it. Finally, OW-Desync’s advantage is on the communication-freshness (peak-age) objective it is built to optimise; on the exploration task it preserves efficiency rather than improving it further, because the frozen explorer is largely insensitive to which teammate is freshest. The analysis also presumes reasonably accurate self-localisation, an essentially static environment, and communication delays that are short relative to the mission: large localisation error corrupts the shared belief irrespective of its age, moving objects add a staleness source that no broadcast schedule can control, and delays comparable to the task duration would leave the stationary age model of Section 3.4 inapplicable. Whether coordination architectures beyond the three planners tested, for example, explicit task-allocation or market-based teams, pay a similar synchrony tax also remains untested.

Several extensions follow naturally. OW-Desync’s activation frequencies are reachable decentrally from local overhearing, which we verify only in simulation here (the hardware demonstration assigns its offsets centrally), so a fully on-robot realisation is the obvious next step. Beyond deciding *when* to broadcast, choosing *which* teammates to refresh (targeting) is a complementary lever, and co-training the policy together with the scheduler could let the explorer exploit freshness it currently ignores. The four-robot WiFi experiments of Section 4.9 reproduce the timing-to-staleness stage on hardware with statistical support (five repeated paired trials on a second fleet, all concordant); task-level travel and overlap statistics on hardware need a larger campaign, roughly twenty pairs for a ten-percent travel effect by our power calculation, under genuinely range-limited, lossy links, which is the next step.

## 7. Conclusions

We studied communication scheduling for bandwidth-limited multi-robot exploration on a frozen learned belief-coupled policy. Empirically, through a decisive phase-only control that holds the per-robot broadcast rate fixed (desynchronisation in fact transmits up to 16% more bytes) and across team-size sweeps, and theoretically via a staleness → overlap bound, we showed that for such a policy communication *synchronisation* is the controllable efficiency cost under scarce bandwidth, a *synchrony tax*: desynchronising broadcast timing recovers efficiency without relying on byte savings, and even simple phase staggering is provably bound-optimal in the symmetric single-medium regime, where an exhaustive scheduler sweep confirms no sophisticated alternative beats it. On a realistic range-limited interference channel with heterogeneous robots, we generalise even staggering to *OW-Desync*, a provably peak-age-optimal overlap-weighted activation policy that subsumes even staggering as its symmetric special case (Theorem 1) and strictly improves communication freshness under heterogeneity while preserving exploration efficiency on the frozen explorer; its schedule is also reachable decentrally with no coordinator. The effect is a property of learned belief-coupled coordination rather than a universal law, disappearing (with at most a small apparent cost) on planners that barely deconflict through the shared belief. Four-robot experiments over real WiFi show that the first stage of the mechanism, from communication timing to peak team staleness, survives a physical channel: at matched rate, even staggering cuts the measured peak by 34–37% across six paired runs on two robot fleets, with five repeated trials giving the stage statistical support (all pairs concordant, paired *t* $p<10^{-7}$), close to the predicted optimum, with the mean essentially unchanged. Repeated paired hardware trials for task-level efficiency are the planned next step.

In one sentence: under scarce bandwidth, broadcast phase is a free coordination resource for belief-coupled multi-robot learning; its benefit has a single staleness mechanism, whose timing stage survives a real channel, appears exactly where coordination runs through the shared belief, and on a symmetric medium is already captured optimally by plain even staggering, with OW-Desync carrying it to the heterogeneous, interference-limited setting.

## Figures and Tables

**Table 1:** Broadcast schedulers at a matched scarce budget (N=8, ≈47–50 kB communication). Coverage and communication are matched; the desynchronised and event-triggered schedules cut travel and overlap. Lower travel/overlap/steps are better.

Scheduler	Coverage	Comm (10^3^ B)	Travel	Overlap	Steps
Periodic (synchronised)	0.966	47	1231.2	0.378	17.7
Random (desynchronised)	0.966	48	1101.2	0.337	15.8
Event-triggered	0.966	50	1124.1	0.340	16.2

**Table 2:** Staggered vs. synchronised periodic at matched per-robot broadcast rate (N=8, n=160 paired episodes per period; negative = de-sync better; paired Wilcoxon). The last column gives the Holm-adjusted travel *p* across the three-period sweep; every period stays significant ($p_{\mathrm{Holm}}<0.05$), including the marginal period 8. The realised byte volume is not held equal: staggered transmits up to 16% *more* (column 2), yet travels and overlaps less, so the gain is not a bandwidth saving.

Period	Comm (kB) Sync → Staggered	Travel Δ	Overlap Δ	*p* (Travel)	$p_{\mathrm{Holm}}$
5	100.0→106.6	−6.4%	−7.7%	$1.5\times 10^{-3}$	$3.0\times 10^{-3}$
8	69.9→79.0	−5.1%	−6.2%	$4.5\times 10^{-2}$	$4.5\times 10^{-2}$
12	53.6→62.0	−9.8%	−13.2%	$1.4\times 10^{-5}$	$4.2\times 10^{-5}$

**Table 3:** Desynchronisation advantage across team size (phase-only control: staggered vs. synchronised periodic at interval 12, matched per-robot broadcast rate; paired Wilcoxon, negative is better). The travel effect is significant for every N≥6.

*N*	Travel Δ	Overlap Δ	*p* (Travel)	*n*
2	+0.9%	−7.4%	0.89	120
4	−5.3%	−13.6%	0.07	120
6	−9.2%	−12.6%	$2\times 10^{-4}$	120
8	−12.9%	−14.4%	$1.6\times 10^{-6}$	120
10	−6.7%	−6.2%	$1\times 10^{-3}$	120
12	−9.7%	−6.7%	$1.8\times 10^{-4}$	120
16	−7.5%	−4.7%	$4\times 10^{-4}$	111
20	−10.2%	−4.5%	$2.5\times 10^{-6}$	102

**Table 4:** Desynchronisation effect by planner backbone (staggered vs. synchronised periodic, N=8, interval 12; negative travel = de-sync better; paired Wilcoxon, *n* as shown; DARS and utility from the three-backbone run, nearest-frontier from the deconfliction pilot). The effect tracks how strongly each planner coordinates through the shared teammate belief. n.s. means not significant.

Planner Backbone	Belief Coupling	Travel Δ	*p* (Travel)	*n*
Nearest-frontier (myopic)	Weak	+3.9%	0.011	120
Utility-frontier	Moderate	−1.2%	0.66 (n.s.)	100
DARS (learned graph-attention)	Strong	−11.3%	$1.8\times 10^{-4}$	100

**Table 5:** The desynchronisation benefit grows with the team’s initial information correlation (N=8, interval 12, staggered vs. synchronised periodic; paired Wilcoxon, n=160). Clustered spawns start with highly correlated beliefs and sensing footprints, dispersed spawns with weakly correlated ones.

Initial Correlation	Travel Δ	Overlap Δ	*p* (Travel)
Dispersed (low)	−6.1%	−8.7%	$3\times 10^{-5}$
Clustered (high)	−15.6%	−13.9%	<$10^{-3}$

**Table 6:** OW-Desync on the frozen explorer under a range-limited channel (N=16, heterogeneous sensing, paired over 231–240 map–seed pairs per cell; travel change, paired Wilcoxon; * p<0.05, ** p<0.01); n.s. means not significant. OW-Desync ties even staggering (preserving exploration efficiency) while both desynchronised schedules keep the synchrony-tax advantage over synchronisation.

Comm. Range	OW-Desync vs. Even	Even vs. Sync	OW-Desync vs. Sync
Full mesh	−1.0% (n.s.)	−4.6% **	−5.6% **
$R_{c}\;=\;30$	+2.5% (n.s.)	−4.1% **	−1.7% *
$R_{c}\;=\;60$	−0.5% (n.s.)	−4.4% **	−4.9% **

**Table 7:** Hardware demonstration: four robots, one trial per condition, all timing measured at a central monitor over real WiFi. Rates and payloads are matched; only the phase differs. Broadcast counts differ because the staggered recording is 30 s shorter. Ideals are the no-jitter values for N=4, T=4 s.

	Synchronised	Staggered (De-Sync)	Ideal
Per-robot period (median)	4.00 s	4.00 s	T=4 s
Per-robot fire rate (per tick)	0.0505	0.0506–0.0519	0.05
Payload per broadcast	272 B	272 B	Equal
Broadcasts per robot	47	39–40	Rate-matched
Received phase offsets (mod *T*)	{0,0.08,0,0.08} s	{0.20,1.06,2.00,3.05} s	{0,1,2,3} s
Peak team staleness	15.8 s	10.5 s (−34%)	16 vs. 10 s
Mean team staleness	8.0 s	7.9 s	NT/2=8 s

**Table 8:** Five repeated paired hardware trials on the second fleet (matched analysis window *W* per pair; peak and mean of the team-staleness sum; broadcasts counted within *W*). The peak falls by 37% in every pair while the mean and the broadcast count are unchanged. n.s. means not significant.

Pair	*W* (s)	Peak τ Sync (s)	Peak τ Stag. (s)	Mean τ Sync/Stag. (s)	Broadcasts Sync → Stag.
1	85.6	15.84	10.02	7.94/8.01	83 → 84
2	73.5	15.93	10.03	7.90/7.95	75 → 72
3	86.7	15.94	10.06	7.91/7.97	87 → 86
4	71.7	15.95	9.89	7.94/7.96	71 → 71
5	84.2	15.80	10.08	7.95/7.98	83 → 83

## Data Availability

The simulation and hardware data and the analysis code supporting this study, together with our scheduling layer (the desynchronised schedulers and evaluation runners), are available from the corresponding author on reasonable request and will be released publicly upon publication. The work builds on the publicly available bandwidth-limited multi-robot exploration framework of [9]; the pretrained policy is used frozen and unmodified.

## Appendix A. Proofs of the Theoretical Results

**Proof of Lemma 1.** Let $s=t-\tau_{ij}\left(t\right)$ be the last delivery step, so ${\hat{p}}_{j}^{\left(i\right)}\left(t\right)=p_{i}\left(s\right)$. By the triangle inequality and the per-step bound,

(A1) $$\begin{array}{cc} e_{ij}\left(t\right) & =∥\sum_{u=s+1}^{t}\left(p_{i}\left(u\right)-p_{i}\left(u-1\right)\right)∥\leq \sum_{u=s+1}^{t}∥p_{i}\left(u\right)-p_{i}\left(u-1\right)∥\\ \; & \leq \left(t-s\right)\;v_{\max}=\tau_{ij}\left(t\right)\;v_{\max}. \end{array}$$

□

**Proof of Proposition 1.** Round-robin partitions L into ⌈L/B⌉ groups of at most *B* links; group *k* is served at steps ≡k(mod⌈L/B⌉). Each link lies in exactly one group, so it is served once every ⌈L/B⌉ steps and its staleness never exceeds ⌈L/B⌉−1. □

**Proof of Proposition 2.** Only contending pairs ($w_{ij}=1$) can co-sense, so non-contending pairs contribute nothing. For a contending pair, by Assumption 1 the expected redundant-sensing contribution is non-decreasing in $e_{ij}$; summing over pairs and bounding by a non-decreasing envelope Φ of the total weighted belief error $\sum_{ij}w_{ij}e_{ij}$ yields the first inequality. Applying Lemma 1 term-by-term ($e_{ij}\leq \tau_{ij}v_{\max}$) and monotonicity of Φ yields the second. □

**Proof of Proposition 3.** Fix a sender *i*. Under a period-*T* schedule with offset $\phi_{i}$, link i→j delivers exactly at the steps $t\equiv \phi_{i}\;\left(\mathrm{mod}\;T\right)$ (delivery depends only on when the sender broadcasts), so its staleness is the period-*T* sawtooth $\tau_{ij}\left(t\right)=\left(t-\phi_{i}\right)\;\mathrm{mod}\;T\in \left\{0,1,\ldots ,T-1\right\}$, identical for every receiver *j*. Hence the team-staleness sum is the superposition of *N* unit-rate sawtooths, $S\left(t\right)=\sum_{ij}w_{ij}\;\tau_{ij}\left(t\right)=\sum_{k}a_{k}\;\left((t-\phi_{k})\;\mathrm{mod}\;T\right)$, where $a_{k}=\sum_{j\neq k}w_{kj}\geq 0$ is the (nonnegative) outgoing contending weight of sender *k*. *Synchronised case.* If all offsets coincide, $\phi_{k}\equiv \phi$, then every sawtooth reaches its maximum T−1 at the same step t≡ϕ−1(modT), so $\max_{t}S\left(t\right)=\left(T-1\right)\sum_{k}a_{k}$, the largest value $\max_{t}S\left(t\right)$ can ever take since each summand is bounded by $a_{k}\left(T-1\right)$. Thus synchronisation maximises the peak. *Even spacing.* Take the symmetric, fully contending case $a_{k}\equiv a$ (the worst case for overlap, all pairs near). Then $S\left(t\right)=a\sum_{k}\left((t-\phi_{k})\;\mathrm{mod}\;T\right)$. As *t* advances one step every non-wrapping term increases by 1 and a term whose sawtooth wraps drops by T−1, so between wraps *S* climbs at rate *N* per step and its peak is attained immediately before some wrap, at an instant $t\equiv \phi_{m}-1\;\left(\mathrm{mod}\;T\right)$. Evaluate *S* at all *N* pre-wrap instants and average. For distinct offsets, pairing the contributions of robots *m* and *j* across the two instants gives $\left((\phi_{m}-\phi_{j}-1)\;\mathrm{mod}\;T\right)+\left((\phi_{j}-\phi_{m}-1)\;\mathrm{mod}\;T\right)=T-2$, so the average pre-wrap value equals $a\left(N(T-1)/2+(T-N)/2\right)$ for *every* assignment with distinct offsets, while coincident offsets only raise it (a coincident pair contributes 2(T−1)>T−2). The maximum is at least the average, so every schedule has $\max_{t}S\left(t\right)\geq a\left(N(T-1)/2+(T-N)/2\right)$. When *N* divides *T* the evenly spaced assignment makes all *N* pre-wrap values equal, attaining this lower bound with equality, so it is exactly optimal; the synchronised assignment attains aN(T−1), the largest value any schedule can reach. For general T≥N, write $\phi_{k}=\left\lfloor \left(k-1\right)T/N\right\rfloor$. For m≠k the offset difference $\phi_{m}-\phi_{k}$ differs from (m−k)T/N by less than 1, so each pairwise term $\left((\phi_{m}-\phi_{k}-1)\;\mathrm{mod}\;T\right)$ differs from its evenly spaced value by less than 1, and each pre-wrap value of S/a differs from the assignment-independent average N(T−1)/2+(T−N)/2 by less than N−1. The balanced peak therefore exceeds the universal lower bound by less than a(N−1), a guarantee no schedule can beat by more than that margin; an exhaustive search over all offset assignments for N≤6, T≤16 in fact finds the balanced assignment exactly optimal in every case. When T<N distinct offsets are infeasible; the peak is then minimised by balancing the senders across the *T* slots (⌊N/T⌋ or ⌈N/T⌉ per slot), which the same assignment achieves, while synchronisation (all robots in one slot) remains the maximiser. Indeed an unbalanced assignment places at least one extra simultaneous reset in some slot and leaves a longer unopposed climb elsewhere, so balancing the slot loads minimises the maximum of the sawtooth superposition. The general weighted and interference-limited case does *not* reduce to even phase-spacing at a fixed rate (a heavy sender may need a different share of the medium), and is handled instead by Theorem 1, which optimises activation *frequencies* rather than phases. Therefore the balanced assignment $\phi_{k}=\left\lfloor \left(k-1\right)T/N\right\rfloor$ minimises $\max_{t}\sum_{ij}w_{ij}\tau_{ij}\left(t\right)$, and by the monotonicity of Φ(·) in Proposition 2 it minimises the overlap envelope. The evenly spaced staggering used throughout is thus the bound-optimal open-loop, zero-side-information schedule, and the synchronised periodic baseline is its worst case. □

**Proof of Theorem 1.** For any policy, let $f_{i}$ be source *i*’s long-run *activation* (transmission-attempt) frequency; the achievable set of *f* is exactly P, since each slot selects one independent set and successful delivery on link *i* occurs with probability $\gamma_{i}$ when *i* transmits, while any point of P is realised by a stationary policy that samples *S* with probability $x_{S}$ (Birkhoff-type decomposition of *f* into independent-set indicators). The *expected* (time-average) peak age of source *i* under a stationary policy with activation frequency $f_{i}$ and per-attempt success probability $\gamma_{i}$ is, by a geometric renewal, $1/(\gamma_{i}f_{i})$, so $A_{p}=\sum_{i}w_{i}/\left(\gamma_{i}f_{i}\right)$, a convex function of *f* on P; minimising it gives $f^{★}$ and the optimal stationary policy. This instantiates the peak-age optimality of stationary policies on interference networks [11] with task weights $w_{i}$ and an average per-slot budget. □

**Proof of Corollary 1.** For complete *G* only one robot transmits per slot, so $P=\{f\geq 0:\sum_{i}f_{i}\leq \min\left(1,B\right)\}$. The objective $\left(w/\gamma \right)\sum_{i}1/f_{i}$ is symmetric and strictly convex; by Jensen (or the KKT conditions) its minimiser over the simplex is uniform, $f_{i}^{★}=\min\left(1,B\right)/N$, which is round-robin with even offsets $\phi_{k}=\left\lfloor \left(k-1\right)T/N\right\rfloor$. Uniform marginals fix only the *expected* peak age; among the schedules that realise them, the even offsets additionally minimise the worst-case peak by Proposition 3. □

**Proof of Proposition 4.** On the simplex $\sum_{i}f_{i}=B$ the stationarity condition $\partial_{f_{i}}\;\sum_{i}\left(w_{i}/\gamma_{i}\right)/f_{i}$=λ gives $f_{i}^{★}\propto \sqrt{w_{i}/\gamma_{i}}$, which is non-uniform exactly when the $w_{i}/\gamma_{i}$ differ. Since $\sum_{i}c_{i}/f_{i}$ (with $c_{i}=w_{i}/\gamma_{i}$) is strictly convex and $f^{\mathrm{RR}}=B/N1$ is feasible but not stationary, $A_{p}\left(f^{★}\right)<A_{p}\left(f^{\mathrm{RR}}\right)$; substituting the two allocations, $A_{p}\left(f^{\mathrm{RR}}\right)/A_{p}\left(f^{★}\right)=\left(\sum_{i}c_{i}\right)/{\left(\sum_{i}\sqrt{c_{i}}\right)}^{2}\cdot N$, which increases with the spread of $\left\{c_{i}\right\}$ (equality iff all $c_{i}$ equal, by Cauchy–Schwarz). When graph constraints beyond the total budget bind, this closed form no longer applies and the optimum is the convex program of Theorem 1. □

**Proof of Corollary 2.** Aggregate the pair weights per sender, $w_{i}=\sum_{j\neq i}w_{ij}$, and bound each $\tau_{ij}\left(t\right)$ by sender *i*’s stationary peak age $\tau_{i}=1/\left(\gamma_{i}f_{i}\right)$; then $\sum_{ij}w_{ij}\tau_{ij}\leq \sum_{i}w_{i}\tau_{i}=A_{p}$ and, by Proposition 2 and the monotonicity of Φ, $E\left[\mathrm{overlap}\right]\leq \Phi \left(v_{\max}A_{p}\right)$ is minimised exactly when $A_{p}$ is, i.e., at $f^{★}$. □
